# LncRNA MALAT1 facilitates BM-MSCs differentiation into endothelial cells and ameliorates erectile dysfunction via the miR-206/CDC42/PAK1/paxillin signalling axis

**DOI:** 10.1186/s12958-024-01240-8

**Published:** 2024-06-25

**Authors:** Longhua Luo, Zixin Wang, Xuxian Tong, Tenxian Xiong, Minggen Chen, Xiang Liu, Cong Peng, Xiang Sun

**Affiliations:** 1 Jiangxi Provincial Key Laboratory of Urinary System Diseases, Department of Urology, The First Affiliated Hospital, Jiangxi Medical College, Nanchang University, No. 17, Yongwai Zheng Street, Nanchang, 330006 China; 2 Nanchang University, No. 999 Xuefu Avenue, Honggutan District, Nanchang City, 330006 Jiangxi Province China

**Keywords:** BM-MSCs, Diabetes mellitus erectile dysfunction, MALAT1, miR-206/CDC42/PAK1/paxillin signalling pathway

## Abstract

**Background:**

Erectile dysfunction (ED) is a common male sexual dysfunction, with an increasing incidence, and the current treatment is often ineffective.

**Methods:**

Vascular endothelial growth factor (VEGFA) was used to treat bone marrow-derived mesenchymal stem cells (BM-MSCs), and their cell migration rates were determined by Transwell assays. The expression of the von Willebrand Factor (vWF)VE-cadherin, and endothelial nitric oxide synthase(eNOS) endothelial markers was determined by qRT‒PCR and Western blot analyses. The MALAT1-induced differentiation of BM-MCs to ECs via the CDC42/PAK1/paxillin pathway was explored by transfecting VEGFA-induced BM-MSC with si-MALAT1 and overexpressing CDC42 and PAK1. The binding capacity between CDC42, PAK1, and paxillin in VEGFA-treated and non-VEGFA-treated BM-MSCs was examined by protein immunoprecipitation. MiR-206 was overexpressed in VEGFA-induced BM-MSC, and the binding sites of MALAT1, miR-206, and CDC42 were identified using a luciferase assay. Sixty male Sprague‒Dawley rats were divided into six groups (*n* = 10/group). DMED modelling was demonstrated by APO experiments and was assessed by measuring blood glucose levels. Erectile function was assessed by measuring the intracavernosa pressure (ICP) and mean arterial pressure (MAP). Penile erectile tissue was analysed by qRT‒PCR, Western blot analysis, and immunohistochemical staining.

**Results:**

MALAT1 under VEGFA treatment conditions regulates the differentiation of BM-MSCs into ECs by modulating the CDC42/PAK1/paxillin axis. In vitro experiments demonstrated that interference with CDC42 and MALAT1 expression inhibited the differentiation of BM-MSCs to ECs. CDC42 binds to PAK1, and PAK1 binds to paxillin. In addition, CDC42 in the VEGFA group had a greater ability to bind to PAK1, whereas PAK1 in the VEGFA group had a greater ability to bind to paxillin. Overexpression of miR-206 in VEGFA-induced BM-MSCs demonstrated that MALAT1 competes with the CDC42 3’-UTR for binding to miR-206, which in turn is involved in the differentiation of BM-MSCs to ECs. Compared to the DMED model group, the ICP/MAP ratio was significantly greater in the three BM-MSCs treatment groups.

**Conclusions:**

MALAT1 facilitates BM-MSC differentiation into ECs by regulating the miR-206/CDC42/PAK1/paxillin axis to improve ED. The present findings revealed the vital role of MALAT1 in the repair of BM-MSCs for erectile function and provided new mechanistic insights into the BM-MSC-mediated repair of DMED.

**Supplementary Information:**

The online version contains supplementary material available at 10.1186/s12958-024-01240-8.

## Introduction

Diabetes-induced erectile dysfunction is called diabetes mellitus erectile dysfunction (DMED) [1]. The incidence of DMED is increasing annually, and it has become an important factor affecting men’s sexual function. However, the underlying mechanisms remain poorly understood. The mechanisms of ED complications in diabetic patients are complex. ED is the result of a complex neurovascular process that involves the integrative synchronized action of the vascular endothelium, smooth muscle, the psychological system, the neuronal system, and the hormonal system [2]. Sildenafil, tadalafil, and phosphodiesterase type 5 (PDE5) inhibitors are commonly used to prevent and control DMED in Western medicine, but they only improve local symptoms [3, 4]. DMED patients are resistant to PDE5 inhibitors [5]. In addition, these medicines have limited use due to their side effects. The maximum single dose of sildenafil administered to men with T2DM results in a mild increase in heart rate and a decrease in blood pressure [6]. Therefore, identifying safer and more effective ED treatments is urgent [7].

BM-MSCs support the healing of injured peripheral nerves, and BM-MSCs accelerate the healing of acute and subacute peripheral nerve injuries [8]. Our previous study confirmed that the transplantation of BM-MSCs effectively improves DMED, and the therapeutic effect of BM-MSCs is further improved by controlling the expression levels of several genes in BM-MSCs [9–11]. Moreover, previous studies have shown that overexpression of MALAT1 in BM-MSCs facilitates differentiation towards endothelial cells (ECs), whereas interference with MALAT1 inhibits the differentiation of BM-MSCs towards ECs. Therefore, the present study will investigate the mechanism by which MALAT1 regulates the differentiation of BM-MSCs towards ECs [11].

Rho GTPase is an important member of the Ras protein superfamily and is involved in the regulation of various cellular events. Rho, Rac, and cell division cycle 42 (CDC42) are the most studied Rho GTPases [12]. One study has shown that upregulation of CDC42 expression and increased CDC42 activity contribute to angiogenesis in cultured endothelial cells in vitro [13]. In addition, elevated CDC42 expression and activation contribute to the ability of BM-MSCs to migrate to the site of skin injury and promote wound healing [14]. However, whether CDC42 contributes to the differentiation of BM-MSCs into endothelial cells has not been reported.

Paxillin is an important cell adhesion factor involved in various cellular events, such as cell proliferation, adhesion, and migration. Paxillin is a downstream target of p21 activated kinase 1 (PAK1), part of a conserved class of serine/threonine kinases [15]. PAK1 induces the phosphorylation of paxillin at several sites, including serine 273 and serine 258 [16, 17]. PAK1 is the major effector protein of CDC42 and Rac1 in the Rho GTPase family, and it participates in endothelial cell proliferation, migration, and angiogenesis by binding to CDC42 and Rac1 to form the Rac1/CDC42/PAK1 complex [18–20]]. Studies have shown that in VEGFA-treated human venous endothelial cells, the expression level of paxillin and its phosphorylation level increase with increasing VEGFA dose, and the expression level of CDC42 also increases [21, 22]. Moreover, several studies have indicated that paxillin is involved in the regulation of endothelial cell proliferation, migration, and angiogenesis [23]. Our previous study revealed that MALAT1 promotes the differentiation of BM-MSCs into endothelial cells by upregulating VEGFA expression [11]. Therefore, the CDC42/PAK1/paxillin signalling pathway may be activated in VEGFA-induced BM-MSCs.

Transplanting stem cells into tissues is a gene therapy for treating DMED-related diseases. The present study investigated the effect of implanting BM-MSCs with modified MALAT1 into the penile tissues of DMED rats for the treatment of ED, and the mechanisms were examined.

## Methods

### Animals and treatment

Male Sprague–Dawley (SD) rats weighing 250–300 g (10 weeks old) were purchased from Beijing Weitong Lihua Laboratory Animal Science and Technology Co., Ltd., and a mating test confirmed that they had normal erectile function. The rats were randomly divided into experimental and control groups after 1 week of free-feeding and water-acclimatization. Rats were injected intraperitoneally with 1% streptozotocin (Solarbio, Beijing, China) to construct a DM rat model, and blood glucose levels were measured after 72 h. Rats with blood glucose levels higher than 16.7 mmol/L were considered successful DM models. Diabetic rats were fed for 8 weeks to develop ED. Rats without penile erection were considered DMED rats, and erectile function was assessed using the apomorphine (APO)-induced erection test (Solarbio, Beijing) [24]. After the DMED model was established in the experimental group of rats, experiments were conducted by implanting PBS, BM-MSCs, BM-MSCs overexpressing MALAT1, or BM-MSCs combined with sildenafil (Solarbio, Beijing). The animal experiments were approved by the Ethics Committee of The First Affiliated Hospital of Nanchang University (CDYFY-IACUC-202312QR023).

### Intracavernosa pressure (ICP) and mean arterial pressure (MAP) assessment

ICP and MAP assessments were performed as previously described [10]. In brief, 3% sodium pentobarbital solution was injected intraperitoneally to anesthetize the rats. The rats were immobilized, and the abdomen was disinfected with iodophor. Two PE50 tubes connected to a pressure-sensing device were placed into the left common carotid artery and the cavernous body of the penis for pressure measurement. The cavernous nerves located in the dorsal aspect of the rat prostate were isolated using a microscopic instrument. Electrical stimulation was performed with a bipolar hook 3–5 mm from the pelvic ganglion for 60 s, resulting in different degrees of penile erection. The physiological pressure signal was transduced by a physiological signal transducer, and the ICP curve was recorded.

### Cell culture and treatment

The rats were sacrificed by cervical dislocation. After isolating the femur, and the bone marrow cavity was exposed and repeatedly washed with low-glucose DMEM (Gibco, Grand Island, NY, USA). The medium containing bone marrow was centrifuged at 1,000 r/min for 10 min, and the supernatant was discarded. The obtained cells were cultured at 37 °C in an incubator containing 5% CO_2_. Cell growth was observed by inverted microscopy. BM-MSCwere treated with 20, 30, 40, or 50 ng/ml VEGFA to induce differentiation towards endothelial cells [11].

### Cell transfection

The cells were transfected as previously described [25]. In brief, MALAT1 or CDC42 cDNA was cloned and inserted into the pcDNA3.1 vector (Invitrogen, Thermo Fisher Scientific). An empty pcDNA3.1 vector was used as a negative control (NC). GenePharma (Shanghai, China) provided the miR-206 mimic/inhibitor and the corresponding control mimic/inhibitor NC, RNA oligonucleotides for MALAT1 knockdown (si-MALAT1), and RNA oligonucleotides for CDC42 knockdown (si-CDC42). Subsequently, the BM-MSCs were transfected using Lipofectamine® 3000 (Invitrogen, USA) before chemical treatment.

### qRT‒PCR

Total RNA from rat cavernosum tissue was extracted by adding TRIzol (Thermo Fisher Scientific), chloroform, and isopropanol according to the manufacturer’s instructions. DNA was removed from the extracted total RNA with a DNA Eraser Buffer kit (TaKaRa, Dalian, China). The obtained RNA was reverse transcribed using a PrimeScript RT Enzyme Mix I kit (TaKaRa). After the reaction was completed, primers were added for PCR amplification. The thermocycler reaction conditions were as follows: 95 °C for 30 s, followed by 40 cycles of 95 °C for 5 s, 55 °C for 30 s, and 72 °C for 30 s. The final results were analysed by the 2^−ΔΔCT^ method.

### Western blot analysis

BM-MSCs in the logarithmic growth phase were washed twice with PBS (Gibco, Grand Island, NY, USA) and lysed with RIPA buffer (Beyotime, Shanghai, China). Total protein (30 µg) was utilized for Western blot analysis. The membranes were incubated overnight at 4 °C with the following primary antibodies: anti-vWF (1:200), anti-VE-cadherin (1:500), anti-eNOS (1:500), anti-MALAT1 (1:500), anti-CDC42 (1:500), anti-PAK1 (1:500), anti-paxillin (1:500), anti-PY31 (1:500), and anti-PY118 (1:500). The membranes were then incubated at room temperature for 1 h with a goat anti-rabbit IgG secondary antibody (1:3,000), and the target bands were visualized and analysed.

Total protein was extracted from the penile cavernosum and lysed with RIPA buffer after grinding in a low-temperature homogenizer, and 60 µg of each sample was utilized for Western blot analysis. The membranes were incubated at 4 °C overnight with the following primary antibodies: anti-vWF (1:200), anti-VE-cadherin (1:500), anti-eNOS (1:500), anti-CDC42 (1:500), anti-PAK1 (1:500), anti-paxillin (1:500), anti-PY31 (1:300), and anti-PY118 (1:500). The membrane was then incubated with a goat anti-mouse IgG secondary antibody (1:3000) at room temperature for 1 h, and the bands were analysed with ImageJ software.

### Coimmunoprecipitation (Co-IP) assay

Coimmunoprecipitation was performed as previously described [26]. Briefly, the collected cells were washed three times with 1× PBS and then lysed on ice in IP lysis buffer containing protease inhibitor cocktail for 1 h. The protein concentration was determined by the BCA method. After preparing the system, 40 L of protein A + G microspheres was added to the extracted protein, followed by incubation at 4 °C for 1 h. The samples were then centrifuged at 14,000×g for 2 min, and the supernatants were removed. Then, 10 L of polyclonal CDC42, PAK1, or paxillin antibody was added, followed by incubation at 4 °C overnight. The samples were centrifuged at 10,000×g for 20 s, and the supernatants were removed. After collecting the microspheres, the samples were washed with PBS, and 50 L of SDS Sampling Buffer was added. The mixture was heated to 85 °C for 10 min and centrifuged at 5,000×g for 10 min. The supernatant was then collected for protein electrophoresis.

### Transwell migration assay

After trypsin digestion, the cell concentration was adjusted to 5 × 10^4^ cells/mL in serum-free DMEM. For the Transwell assay, 200 µL of cell suspension was added to the upper chamber, and DMEM containing10% FBS was added to the lower chamber. The chamber was removed after 24 h, and the cells at the bottom of the upper chamber were removed with a cotton swab. The cells were fixed with 4% paraformaldehyde for 15 min, washed with PBS, stained with 0.5% crystal violet for 15 min. After washing, the cells were imaged under a microscope, and five fields of view were randomly selected for counting. The results of three replicate experiments were analysed statistically analysed.

### Luciferase assay

Wild-type and mutant MALAT1 (with a mutated miR-206 binding site) were cloned and inserted into the pmirGLO dual luciferase vector (GenePharma). BM-MSCs were cotransfected with wild-type pmirGLO-MALAT1 (or mutant) and miR-206 mimics (or negative controls) using Lipofectamine 2000. Similarly, dual-luciferase reporter plasmids containing CDC42-WT and CDC42-MUT were constructed. BM-MSCs were cotransfected with miR-206 mimics and luciferase reporter plasmids or their corresponding empty vector using Lipofectamine 2000. At 48 h after transfection, luciferase activity was analysed using a dual-luciferase reporter kit (Promega, USA).

### Immunohistochemistry

The 10% formaldehyde-fixed tissues were dehydrated by an automatic dehydrator, embedded, and sectioned. The sections were deparaffinized, immersed in 3% methanol hydrogen peroxide for 10 min, and washed three times with PBS (5 min each wash). The sections were heated to boiling in citrate buffer, washed with PBS, and immersed in blocking solution for 20 min. Primary CDC42 and PAK1 were then added and incubated overnight. Biotinylated secondary antibody was added dropwise, and the sections were incubated at 37°C for 30 min. After wash three times with PBS, the sections were stained with 3,3’-diaminobenzidine (DAB). The sections were sealed with clear gum, and photomicrographs were acquired under a light microscope. The results were analysed using Image-Pro Plus 6.0.

### Statistical analysis

Each assay was performed three times. The data were analysed by SPSS 22.0 statistical software (IBM, Armonk, NY, USA) and are expressed as the mean ± standard deviation. Independent sample *t* tests and one-way ANOVA were used to detect differential expression of the indicators. *P* < 0.05 was considered to indicate statistical significance.

## Results

### DMED-treated rats exhibit a decrease in the expression of endothelial marker-related proteins in cavernous tissue

**Fig. 1:**
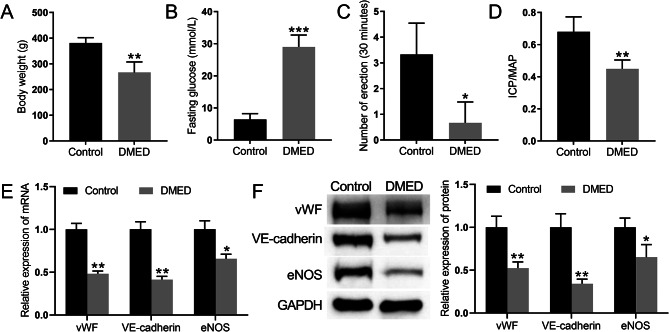
DMED-treated rats exhibit a decrease in the expression of endothelial marker-related proteins in cavernous tissue. (**A**) Body weight of the rats. (**B**) Fasting blood glucose levels of the rats. (**C**) APO experiment to assess erectile function in the rats. (**D**) ICP/MAP ratio of the rats. (**E**) The expression of the vWF, VE-cadherin, and eNOS endothelial markers in the spongiotic tissues of the rats was detected by qRT‒PCR. (**F**) The protein expression of the vWF, VE-cadherin, and eNOS endothelial markers in the spongiotic tissues of the rats was detected by Western blot analysis. *P < 0.05, **P < 0.01, and **P < 0.001 vs. Control

There were no significant differences in baseline body weight or fasting glucose between the groups before modelling. After injecting STZ, the fasting glucose level significantly increased, and the body weight was significantly lower in the STZ group than in the control group (Fig. 1A and B). The number of erections induced by APO in DMED rats was significantly lower than that in the control group (Fig. 1C), and the ICP/MAP ratio was lower in the DMED group than in the control group (Fig. 1D). Changes in the expression of the von Willebrand Factor (vWF), VE-cadherin and endothelial nitric oxide synthase(eNOS) endothelial markers in the spongiotic tissues of rats were detected by qRT‒PCR and Western blot analyses. The expression levels of vWF, VE-cadherin, and eNOS were significantly lower in DMED rats than that in control rats (Fig. 1E and F). Together, the results showed that the DMED rat model was successfully established.

### MALAT1 and CDC42 functions in VEGFA-induced differentiation of BM-MSCs to ECs

**Fig. 2:**
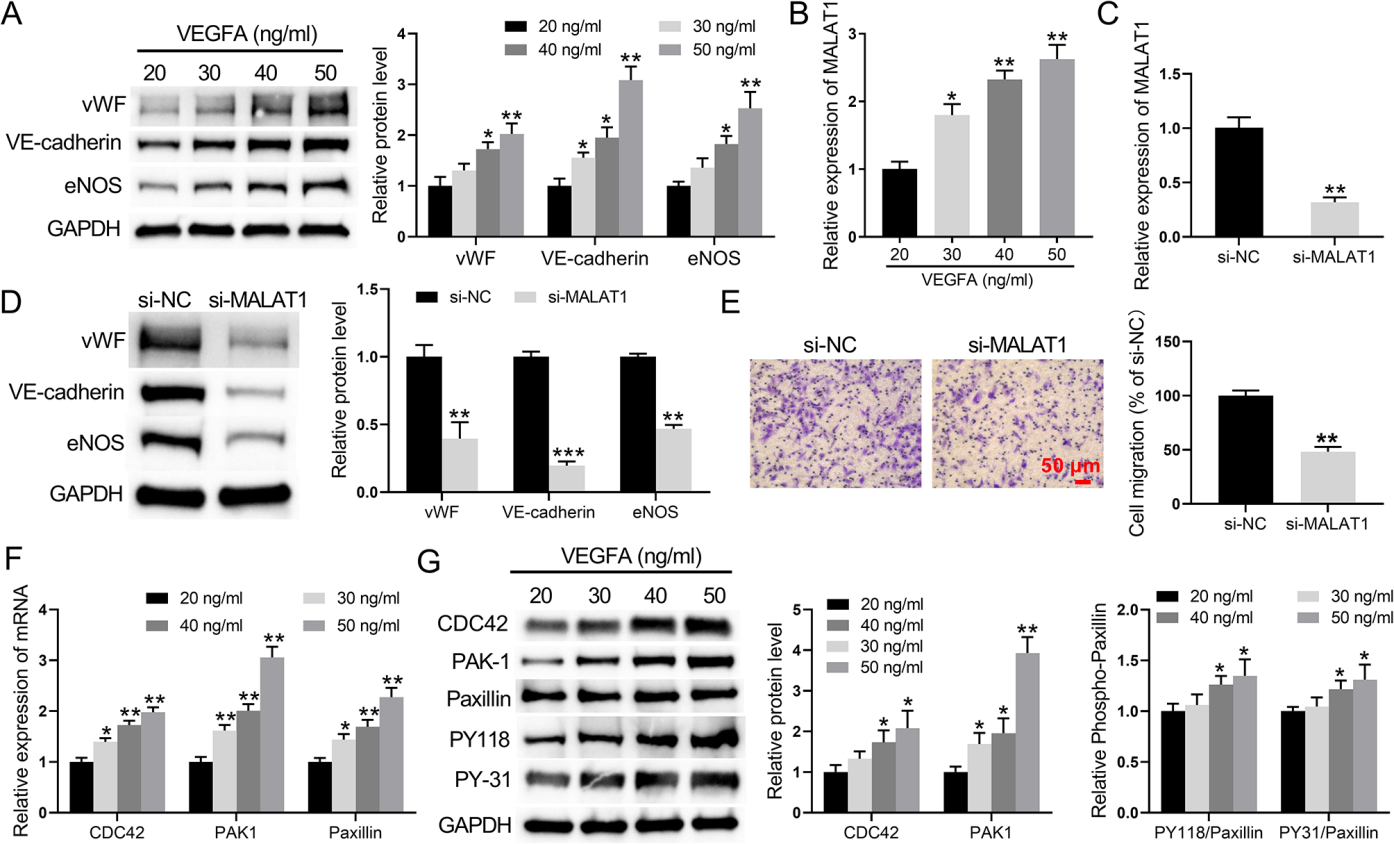
MALAT1 is involved in VEGFA-induced differentiation of BM-MSCs to endothelial cells. (**A**) Western blot analysis was used to detect the protein expression levels of vWF, VE-cadherin, and eNOS in BM-MSCs induced to differentiate into endothelial cells by treatment with different doses of VEGFA for 7 days. (**B**) qRT‒PCR was used to measure the levels of MALAT1 gene expression in BM-MSCs induced to differentiate into endothelial cells after treatment with different doses of VEGFA for 7 days. (**C**) qRT‒PCR was used to detect changes in MALAT1 gene expression after transfection of si-MALAT1 into VEGFA-treated BM-MSCs. (**D**) Western blot analysis was used to detect changes in vWF, VE-cadherin, and eNOS protein expression after transfection of si-MALAT1 into VEGFA-treated BM-MSCs. (**E**) Transwell assays were used to detect the migratory capacity of VEGFA-induced BM-MSCs. (**F**) qRT‒PCR was used to measure the levels of CDC42, PAK1, and paxillin gene expression in BM-MSCs induced to differentiate into endothelial cells by treatment with different doses of VEGFA for 7 days. (**G**) Western blot analysis was used to detect the protein expression levels of CDC42, PAK1, paxillin, and phosphorylated paxillin in BM-MSCs induced to differentiate into endothelial cells by treatment with different doses of VEGFA for 7 days. *P < 0.05, **P < 0.01, and ***P < 0.001 20 ng/mL group or si-NC

After different doses of VEGFA induced the differentiation of BM-MSCs to ECs for 7 days, the protein expression levels of the vWF, VE-cadherin, and eNOS cellular endothelial markers gradually increased with increasing VEGFA dose (Fig. 2A). qRT‒PCR revealed that MALAT1 gene expression gradually increased with increasing VEGFA dose (Fig. 2B). Based on the above experiments, BM-MSCs were differentiated into ECs with 50 ng/ml VEGFA for 7 days for the subsequent experiments. qRT‒qPCR analysis showed that MALAT1 expression was significantly lower in the si-MALAT1-transfected group than in the si-NC-transfected group (Fig. 2C). As shown in Fig. 2D, the expression of vWF, VE-cadherin, and eNOS was downregulated in cells transfected with si-MALAT1. Transwell migration assays indicated that there were fewer migrating BM-MSCs in the siMALAT1 group than in the si-NC group (Fig. 2E).

**Fig. 3:**
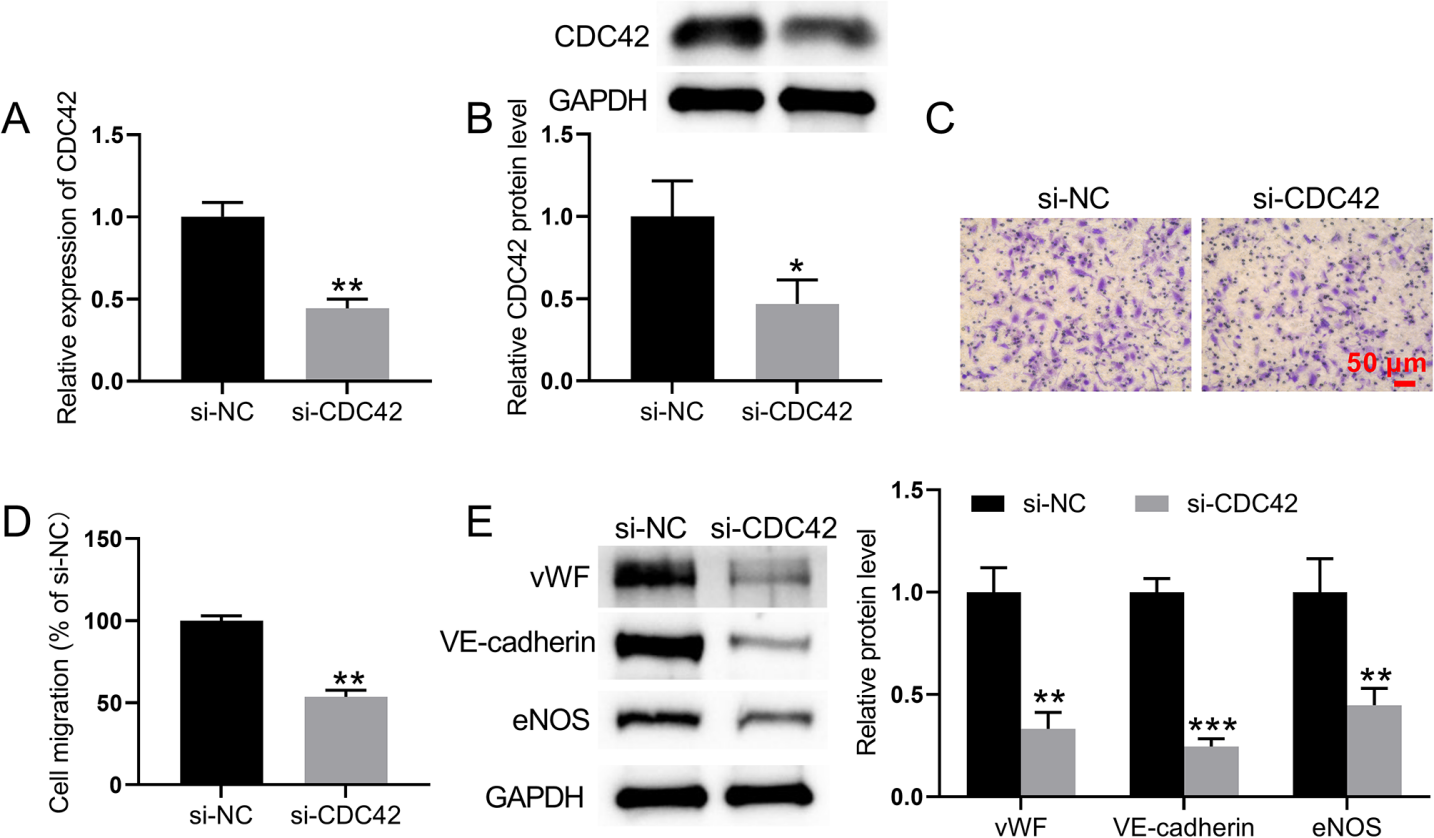
CDC42 is involved in VEGFA-induced differentiation of BM-MSCs to endothelial cells. (**A**) qRT‒PCR was performed to detect the CDC42 mRNA expression level. (**B**) CDC42 protein expression levels were detected by Western blot analysis. (**C-D**) Transwell assays were used to detect the migratory capacity of VEGFA-treated BM-MSCs. (**E**) The expression levels of the vWF, VE-cadherin, and eNOS cellular endothelial markers were detected by Western blot analysis. ^*^P < 0.05 and ^**^P < 0.01 vs. the si-NC group

qRT‒PCR and Western blot analyses were used to examine the impact of different doses of VEGFA on the levels of CDC42, PAK1, paxillin, and paxillin phosphorylation (PY118 and PY-31) in BM-MSCs. After different doses of VEGFA induced the differentiation of BM-MSCs to ECs, the mRNA levels of the CDC42, PAK1, and paxillin cellular endothelial markers gradually increased with increasing VEGFA dose (Fig. 2F). Moreover, the protein expression levels of CDC42, PAK1, and phosphorylated paxillin (PY118 and PY-31) gradually increased with increasing VEGFA dose (Fig. 2G). VEGFA (50 ng) treatment was used to interfere with CDC42 expression in BM-MSCs. qRT‒PCR and Western blot analyses revealed that the expression level of CDC42 was significantly lower in the si-CDC42-transfected group than in the si-NC-transfected group (Fig. 3A and B). Compared to cells transfected with si-NC, Transwell assays showed that cell migration was significantly reduced after transfecting cells with si-CDC42 after VEGFA treatment of BM-MSCs (Fig. 3C and D). Compared to cells transfected with si-NC, the expression levels of vWF, VE-cadherin, and eNOS were downregulated in cells transfected with si-CDC42 (Fig. 3E). Taken together, these data indicated that interference with CDC42 and MALAT1 expression inhibits the differentiation of BM-MSCs to ECs and the expression of endothelial marker-related proteins.

### MALAT1 regulates the differentiation of BM-MSCs into ECs under VEGFA treatment via CDC42/PAK1/paxillin axis

**Fig. 4:**
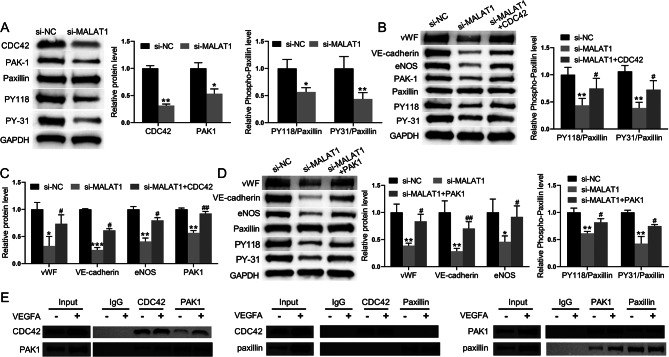
MALAT1 regulates the differentiation of BM-MSCs into ECs under VEGFA treatment via CDC42/PAK1/paxillin axis. (**A**) Western blot analysis was used to measure the levels of CDC42, PAK1, paxillin, and paxillin phosphorylation in VEGFA-induced BM-MSCs. (**B-C**) Western blot analysis was used to detect the expression levels of PAK1, paxillin phosphorylation, vWF, VE-cadherin, and eNOS in VEGFA-induced BM-MSCs. (**D**) Western blot analysis was used to detect the expression levels of PAK1, paxillin, paxillin phosphorylation, vWF, VE-cadherin, and eNOS in VEGFA-induced BM-MSCs. (**E**) Protein immunoprecipitation was used to detect the binding capacity between CDC42, PAK1, and paxillin in VEGFA-treated and non-VEGFA-treated BM-MSCs. *P < 0.05, **P < 0.01, and ***P < 0.001 vs. the si-NC group; ^#^*P* < 0.05 and ^##^*P* < 0.01 vs. the si-MALAT1 group

To analyse whether MALAT1 regulates the differentiation of BM-MSCs into endothelial cells through the CDC42/PAK1/paxillin signalling pathway, VEGFA-induced BM-MSCs were transfected with si-MALAT1. The protein expression of CDC42, PAK1, and phosphorylated paxillin (PY118 and PY-31) in VEGFA-induced BM-MSCs was significantly reduced after transfection with si-MALAT1 (Fig. 4A). MALAT1 was then inhibited while overexpressing CDC42 in VEGFA-induced BM-MSCs. The expression levels of PAK1, phosphorylated paxillin, vWF, VE-cadherin, and eNOS were significantly lower in the si-MALAT1-transfected group than in the si-NC-transfected group. In contrast, the expression levels of PAK1, phosphorylated paxillin, vWF, VE-cadherin, and eNOS were significantly upregulated in VEGFA-induced BM-MSCs with simultaneous CDC42 overexpression and MALAT1 deficiency compared to VEGFA-induced BM-MSCs with only MALAT1 deficiency (Fig. 4B and C). Finally, MALAT1 expression was disrupted and PAK1 was overexpressed in VEGFA-induced BM-MSCs. The protein expression levels of PAK1, phosphorylated paxillin, vWF, VE-cadherin and eNOS were significantly lower in the VEGFA-induced BM-MSCs transfected with si-MALAT1 than in those transfected with si-NC; however, overexpression of PAK1 reversed this effect (Fig. 4D). The binding capacity between CDC42, PAK1, and paxillin in VEGFA-treated and non-VEGFA-treated BM-MSCs was examined by protein immunoprecipitation. CDC42 was demonstrated to bind to PAK1, while PAK1 was demonstrated to bind to paxillin. In addition, CDC42 in the VEGFA group had a greater ability to bind to PAK1. Similarly, PAK1 in VEGFA-treated cells had a greater ability to bind to paxillin (Fig. 4E). Therefore, these findings indicated that MALAT1 conditions regulates the differentiation of BM-MSCs into ECs under VEGFA treatment by modulating the CDC42/PAK1/paxillin axis.

### Mechanism by which MALAT1 regulates the differentiation of BM-MSCs into ECs

**Fig. 5:**
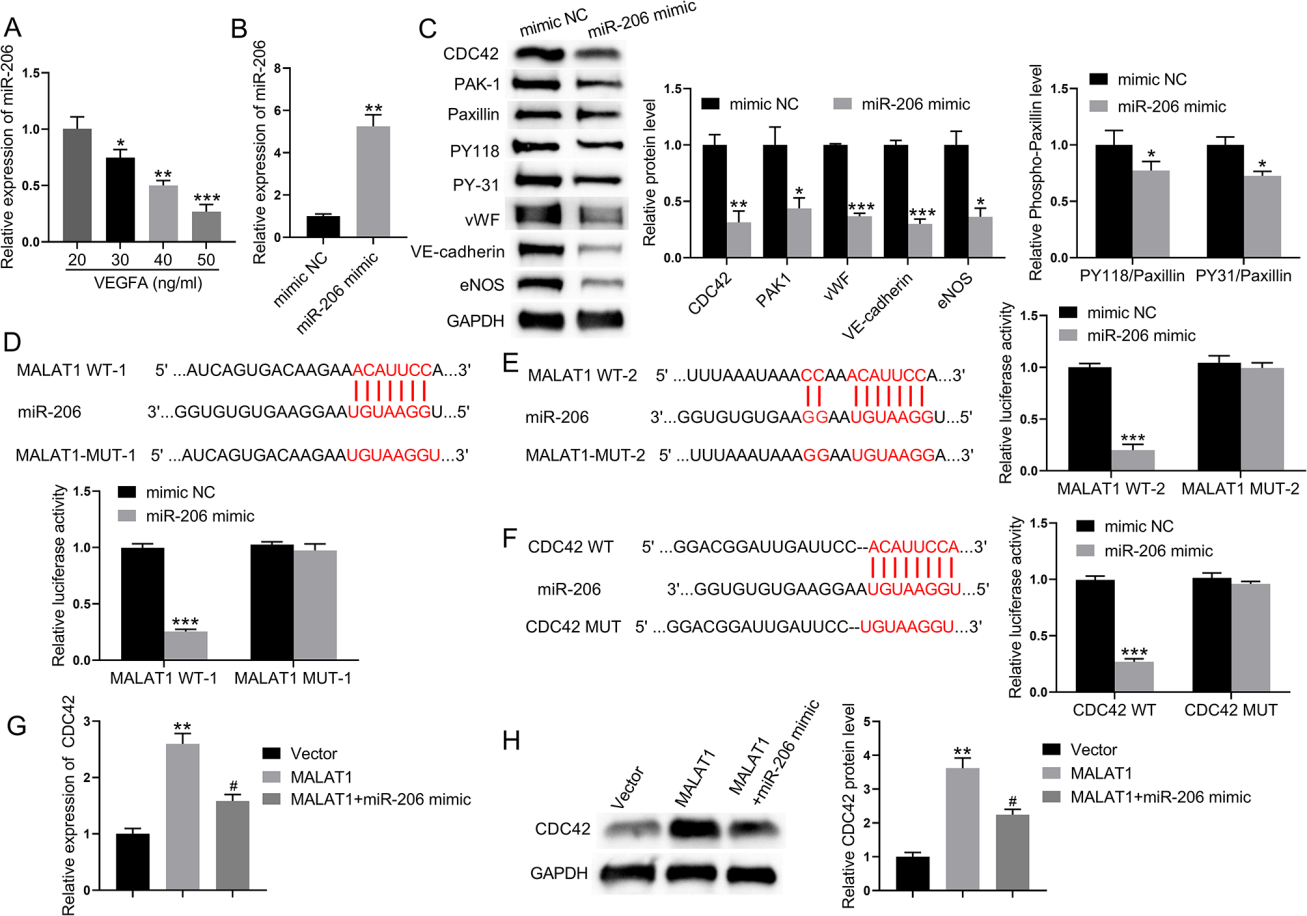
Mechanism by which MALAT1 regulates the differentiation of BM-MSCs into endothelial cells. (**A**) qRT‒PCR was used to detect changes in miR-206 expression in VEGFA-induced BM-MSCs. (**B**) qRT‒PCR was performed to detect the miR-206 expression level. (**C**) Western blot analysis was used to detect the protein expression levels of PAK1, paxillin, paxillin phosphorylation, vWF, VE-cadherin, and eNOS. (**D-E**) Schematic representation of the two binding sites of MALAT1 and miR-206. A luciferase assay was used to detect the binding between the two binding sites of MALAT1 and miR-206. (**F**) Schematic diagram of the CDC42 3’UTR and miR-206 binding site. A luciferase assay was conducted to detect the binding between the CDC42 3’UTR and the miR-206 binding site. (**G**) The CDC42 mRNA level in VEGFA-induced BM-MSCs was determined by qRT‒PCR. (**H**) CDC42 protein expression in VEGFA-induced BM-MSCs was measured by Western blot analysis. *P < 0.05, **P < 0.01, and ***P < 0.001 vs. the 20 ng/mL VEGFA group or mimic NC/vector group. ^#^*P* < 0.05 vs. the MALAT1 group

BM-MSCs were treated with VEGFA (20, 30, 40, or 50 ng/ml) to induce the differentiation of BM-MSCs to ECs in vitro, and qRT‒PCR was used to detect miR‒206 expression in BM‒MSCs. The expression of miR-206 in VEGFA-induced BM-MSCs was the lowest at 50 ng/ml VEGFA (Fig. 5A). Next, miR-206 was overexpressed in BM-MSCs induced with 50 ng/ml VEGFA. qRT-PCR result revealed that the expression level of miR-206 was significantly increased in the miR-206 mimic group (Fig. 5B). Western blot analysis showed that the expression of PAK1, phosphorylated paxillin, vWF, VE-cadherin, and eNOS were decreased in VEGFA-induced BM-MSCs transfected with the miR-206 mimic (Fig. 5C). To verify the interaction between MALAT1 and miR-206, as well as between the CDC42 3’UTR and miR-206, a luciferase reporter assay was performed. The luciferase activity was significantly decreased in VEGFA-induced BM-MSCs in the presence of the miR-206 mimic, MALAT1 WT-1, and MALAT1 WT-2 (Fig. 5D and E). Moreover, the luciferase activity was reduced in VEGFA-induced BM-MSCs in the presence of the miR-206 mimic and CDC42 WT (Fig. 5F). These results indicated that MALAT1 and the CDC42 3’UTR interact with miR-206. Finally, MALAT1 and miR-206 were overexpressed in BM-MSCs induced with 50 ng/ml VEGFA. Compared to the control group, the expression level of CDC42 was significantly greater in the MALAT1 overexpression group. In addition, the expression level of CDC42 was significantly lower in the simultaneous MALAT1 and miR-206 overexpression group than in the MALAT1 overexpression group, but it was not lower than the CDC42 expression level in the control group (Fig. 5G and H). These data indicated that MALAT1 competes with the CDC42 3’-UTR for binding to miR-206, which in turn is involved in the differentiation of BM-MSCs to ECs.

### Involvement of MALAT1-overexpressing BM-MSCs in the repair of erectile function in DMED rats

**Fig. 6:**
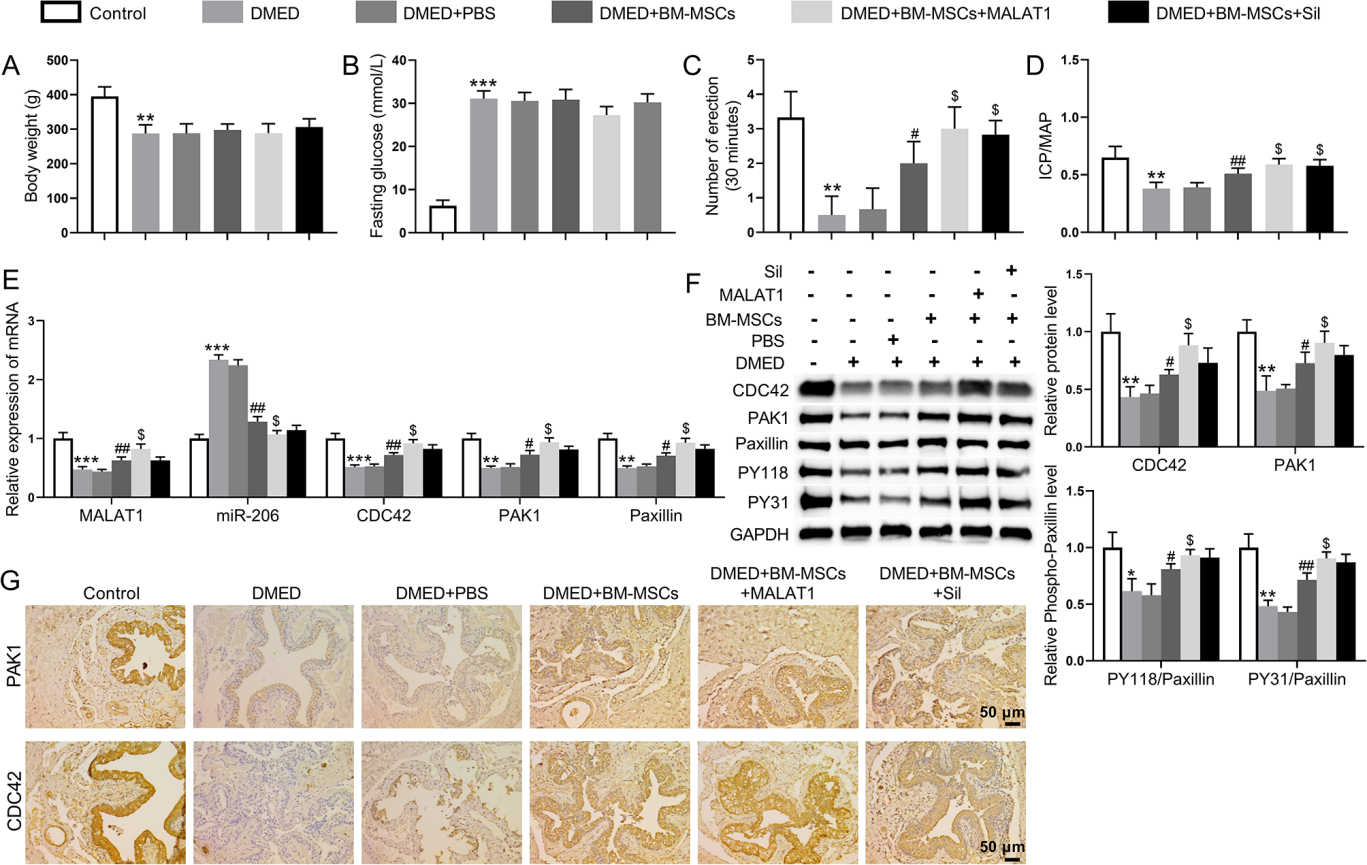
Repair effect of MALAT1 on erectile function in DMED rats. (**A**) Changes in the body weight of the rats in each group. (**B**) Changes in fasting blood glucose in the rats of all the groups. (**C**) The APO assay was used to assess the erectile function of the rats in each group. (**D**) ICP/MAP ratio of the rats in each group. (**E**) qRT‒PCR was used to detect the levels of the MALAT1, miR-206, CDC42, PAK1, and paxillin genes in the penile corpus cavernosum tissues of the rats in each group. (**F**) Western blot analysis was used to detect the protein levels of CDC42, PAK1, paxillin, and paxillin phosphorylation in the penile corpus cavernosum tissues of the rats in each group. (**G**) Representative images of CDC42 and PAK1 immunohistochemistry in rat cavernosum tissues in each group. **P < 0.01, ***P < 0.001 vs. the control group; ^#^*P* < 0.01 and ^##^*P* < 0.01 vs. the DMED + PBS group; ^$^*P* < 0.05 vs. the DMED + BM-MSCs group

Sixty male SD rats were randomly divided into the following six treatment groups (*n* = 10/group): control group, DMED group, DMED + PBS group, BM-MSC group, MALAT1-overexpressing BM-MSC group, and BM-MSC combined with sildenafil group. Compared to the control group, the changes in body weight were significantly lower and the fasting blood glucose levels were significantly higher in the other five groups. However, treatment with BM-MSCs, MALAT1-overexpressing BM-MSCs, or BM-MSCs combined with sildenafil had no significant effect on body weight or blood glucose in in DMED rats (Fig. 6A and B). APO experiments were performed to assess erectile function in each group. The number of erections induced by APO in DMED rats and DMED rats treated with PBS was significantly lower than that in the control group. However, DMED rats treated with BM-MSCs, MALAT1-overexpressing BM-MSCs, or BM-MSCs combined with sildenafil exhibited an increase in the number of erections (Fig. 6C). In addition, the ICP/MAP ratio was lower in the DMED group and the DMED + PBS group than in the control group. Compared to the DMED model group, the ICP/MAP ratio was significantly greater in the three BM-MSC treatment groups (Fig. 6D).

qRT‒PCR was used to detect the mRNA levels of the MALAT1, miR‒206, CDC42, PAK1, and paxillin genes in the rat penile corpus cavernosum tissues in each group. The mRNA expression levels of MALAT1, CDC42, PAK1, and paxillin were significantly lower in DMED rats than in control rats. Treatment of DMEM rats with BM-MSCs significantly upregulated the expression levels of MALAT1, CDC42, PAK1, and paxillin compared to untreated DMED rats. The expression levels of miR-206 were increased in DMED rats, but this effect was reversed after treatment with various BM-MSCs (Fig. 6E). The levels of CDC42, PAK1, paxillin, and paxillin phosphorylation were detected by Western blot analysis. The expression of CDC42, PAK1, and phosphorylated paxillin (PY31 and PY118) was lower in the DMED group than in the control group. However, treatment of DMED rats with various BM-MSCs significantly upregulated the expression of CDC42, PAK1, and paxillin phosphorylation (PY31 and PY118) compared DMED rats (Fig. 6F). Finally, the expression of CDC42 and PAK1 was detected by immunohistochemistry. There were more CDC42- and PAK1-positive cells expressed in the control group compared to the other groups, and CDC42 was mainly distributed in perivascular endothelial cells. The mean optical density values of CDC42 and PAK1 in the DMED group were significantly lower than those in the control group. After treatment of DMED rats with various BM-MSCs, the mean optical densities of CDC42 and PAK1 were significantly greater than those in the DMED group (Fig. 6G). Together, these results indicated that MALAT1-overexpressing BM-MSCs have significant therapeutic effects on DMED rats.

## Discussion

In recent years, with the development of the social economy and changes in individuals’ lifestyles, a high-fat diet and lack of exercise have led to an increasing number of individuals suffering from DM. The increasing prevalence of DM and the younger age of patients are more likely to induce ED, which has become an important factor affecting the health of men’s lives and the harmony of their families, thus attracting attention from sociologists and medical researchers [27]. Medication is a common treatment for ED, but the therapeutic effect is not satisfactory. The use of mesenchymal stem cells for ED treatment is promising, and many studies have been conducted on this topic [28, 29]. However, more studies are needed before this approach is feasible and effective [30, 31]. The present study demonstrated that treatment with MALAT1-overexpressing BM-MSCs significantly attenuates ED in rats, suggesting that MALAT1 plays an important role in improving ED.

MALAT1 is a lncRNA that is highly expressed in lung cancer tissues and has been reported to be associated with tumour cell proliferation and metastasis [32]. However, the role of MALAT1 in erectile function and mesenchymal stem-cell differentiation remains unclear. Our laboratory previously reported that the expression level of MALAT1 in rat penile tissues is significantly increased after transplantation of BM-MSCs, and interference with MALAT1 expression in BM-MSCs significantly reduces the treatment effect of BM-MSCs. The present study suggested that MALAT1 upregulates CDC42 levels by inhibiting miR-206 expression, which promotes PAK1 expression and induces paxillin phosphorylation, thereby contributing to the differentiation of BM-MSCs into endothelial cells.

MicroRNAs are noncoding RNAs that have a length of only 19–30 nucleotides, and they can degrade mRNAs or inhibit their expression through translational repression [33, 34]. In recent years, microRNAs have been found to play a wide range of physiological regulatory roles [35]. In the Rho family, CDC42 is a protein with GTPase activity that is involved in various physiological processes, including cell division, intracellular transport, gene transcription, cell cycle, cell growth, and cell stability [36–38]. The CDC42 3’-UTR has a binding site for miR-206. Overexpression of miR-206 in VEGFA-treated BM-MSCs downregulated the CDC42 expression levels. CDC42 can activate downstream proteins, such as PAKs, ACK1, IQGAPs, and P13Ks, which are closely related to cell invasion, migration, proliferation, and neovascularization [39, 40]. P21-activated kinase-1 (PAK1) is an important effector protein of CDC42 and is closely related to cell motility [41]. Several studies have indicated that paxillin is involved in the regulation of endothelial cell proliferation, migration, and angiogenesis [42, 43]. In the present study, PAK1 expression was significantly elevated in cells with simultaneous overexpression of CDC42 and inhibition of MALAT1 compared to cells with only MALAT1 inhibition. The expression levels of PY118 and PY31 were significantly higher in cells with simultaneous overexpression of PAK1 and inhibition of MALAT1 than in cells with only MALAT1 inhibition.

The present results showed that MALAT1-overexpressing BM-MSCs effectively improve DMED in rats. MALAT1 upregulates CDC42 levels by inhibiting miR-206 expression, which promotes PAK1 expression, induces the phosphorylation of paxillin, and upregulates the expression of the vWF, VE-cadherin, and eNOS endothelial markers, thereby contributing to the differentiation of BM-MSCs to ECs and ultimately improving DMED in rats.

## Conclusions

Overall, the present study demonstrated that MALAT1-overexpressing BM-MSCs effectively ameliorate DM-induced erectile dysfunction in rats. MALAT1 upregulates CDC42 levels by inhibiting miR-206 expression, which promotes PAK1 expression and induces the phosphorylation of paxillin, thereby contributing to the differentiation of BM-MSCs into ECs and ultimately ameliorating DMED in rats.

### Electronic supplementary material

Below is the link to the electronic supplementary material.

Supplementary Material 1

## Data Availability

No datasets were generated or analysed during the current study.

## Declarations

### Animal research

The animal experiments were approved by the Ethics Committee of The First Affiliated Hospital of Nanchang University (CDYFY-IACUC-202312QR023).

## Abbreviations

APO: apomorphine
BM-MSCs: bone marrow-derived mesenchymal stem cells
CDC42: cell division cycle 42
DAB: 3, 3’-Diaminobenzidine
DMED: diabetes mellitus erectile dysfunction
ECs: endothelial cells
ED: erectile dysfunction
eNOS: endothelial nitric oxide synthase
ICP: intra cavernosa pressure
MAP: mean arterial pressure
PAK1: p21 activated kinase 1
PDE5: phosphodiesterase type 5
vWF: von Willebrand Factor
