# Supplementary material for: LncRNA MALAT1 facilitates BM-MSCs differentiation into endothelial cells and ameliorates erectile dysfunction via the miR-206/CDC42/PAK1/paxillin signalling axis
Source: Reprod Biol Endocrinol. 2024 Jun 25;22:74. doi: 10.1186/s12958-024-01240-8 (PMC11197369; doi:10.1186/s12958-024-01240-8)

Figure 1- 1F
eNOs

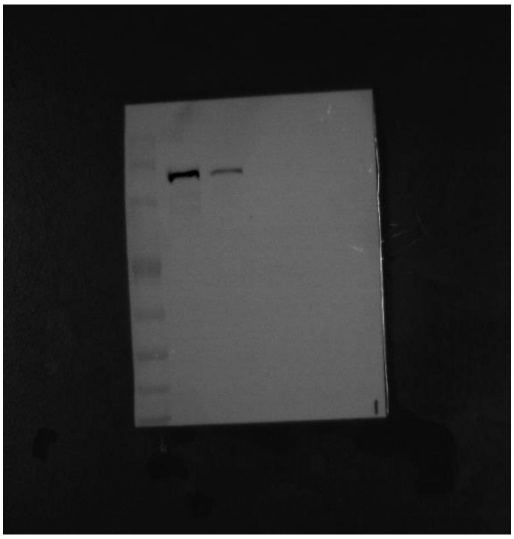

GAPDH

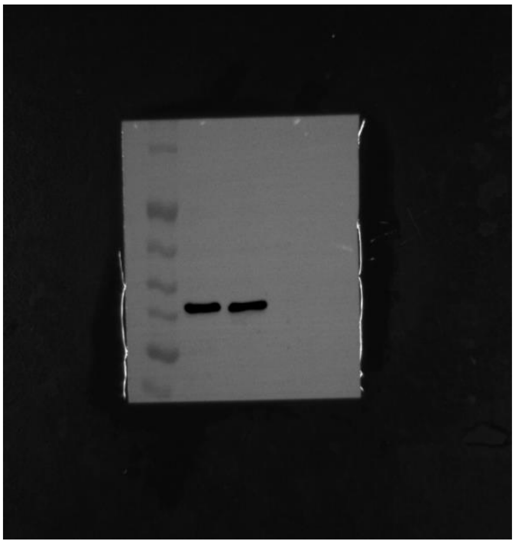

VE-cadhein

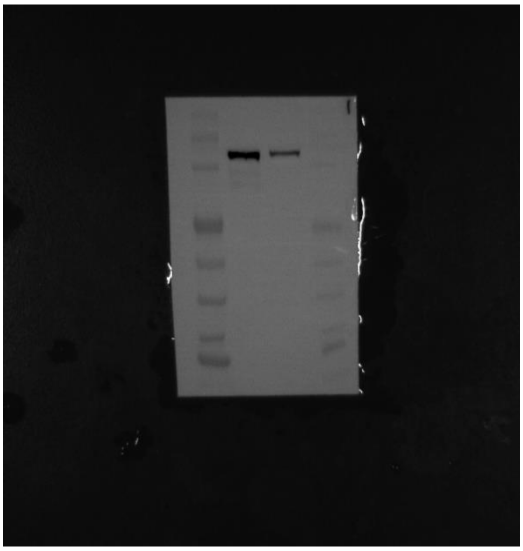

vWF

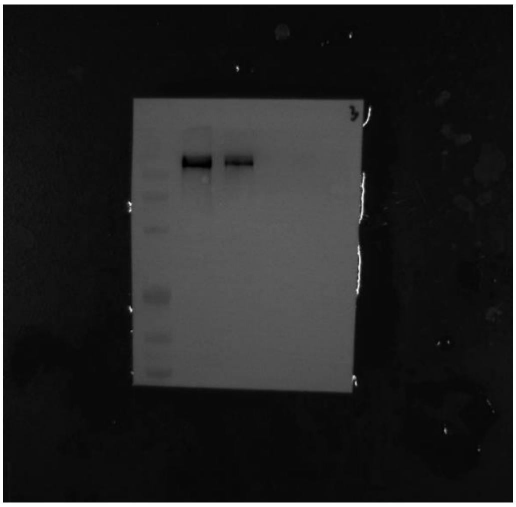

Figure 2-2A

CDC42

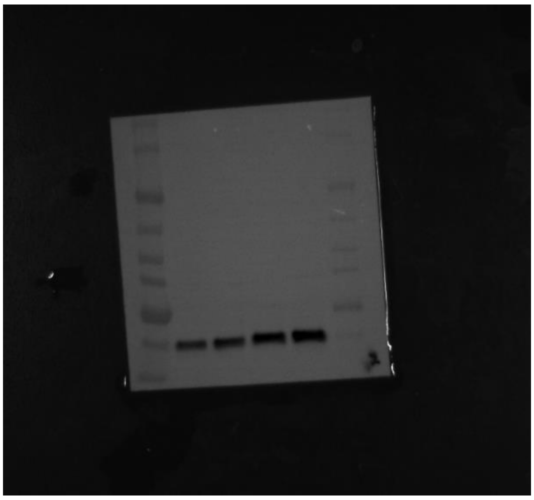

GAPDH

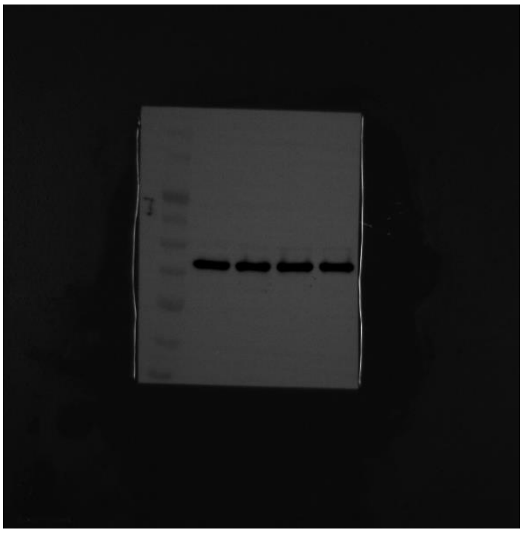

PAK1

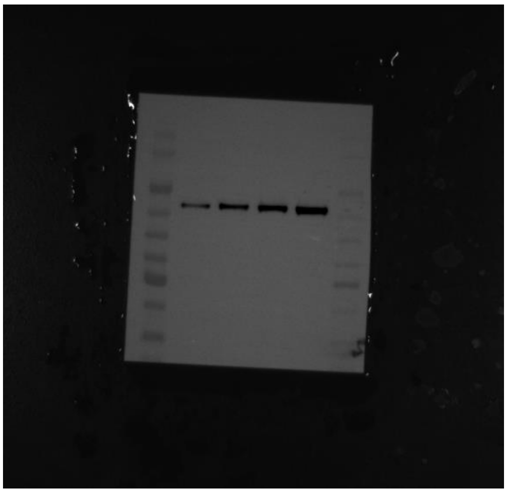

Paxillin

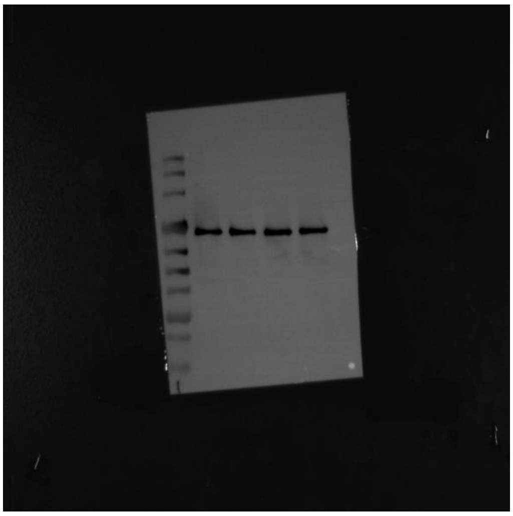

PY-31

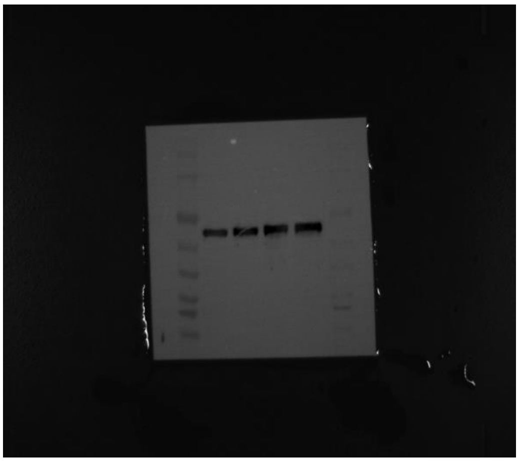

PY118

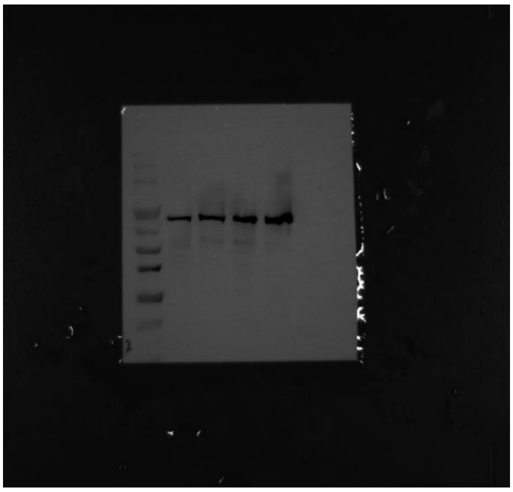

Figure 2-2D

eNOs

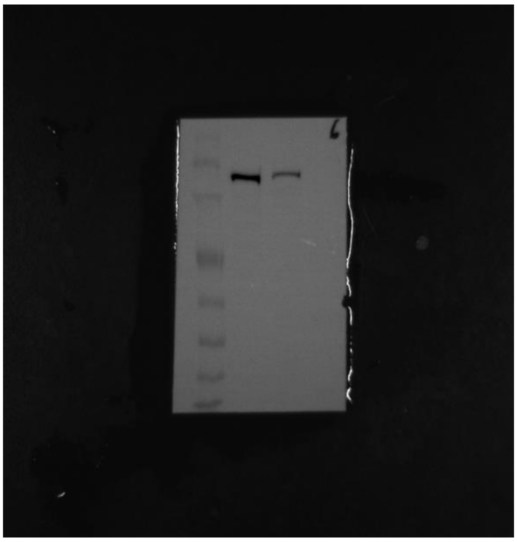

GAPDH

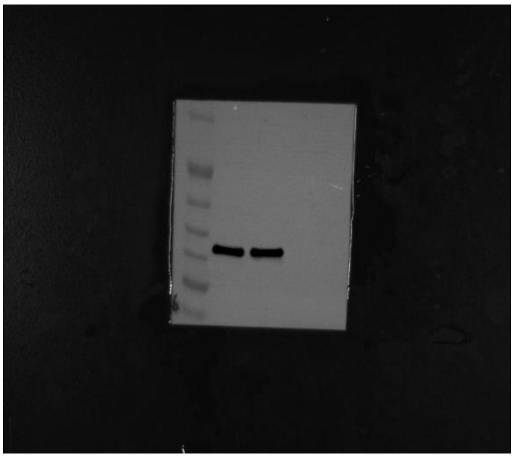

VE-cadhein

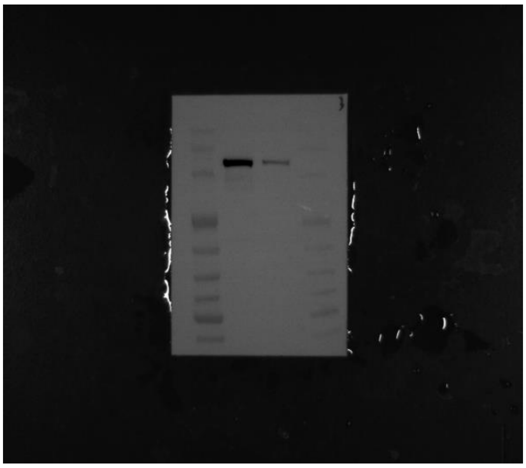

vWF

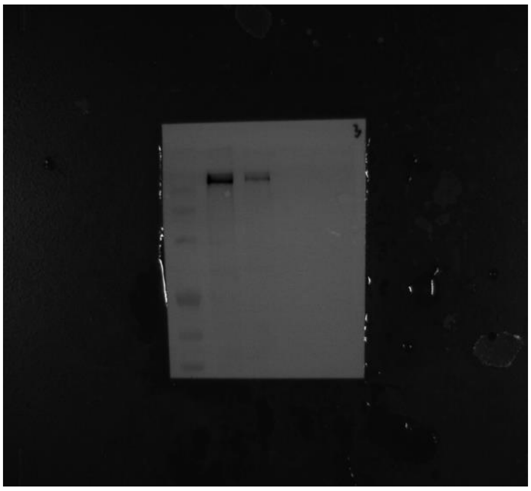

Figure 2-2G
CDC42

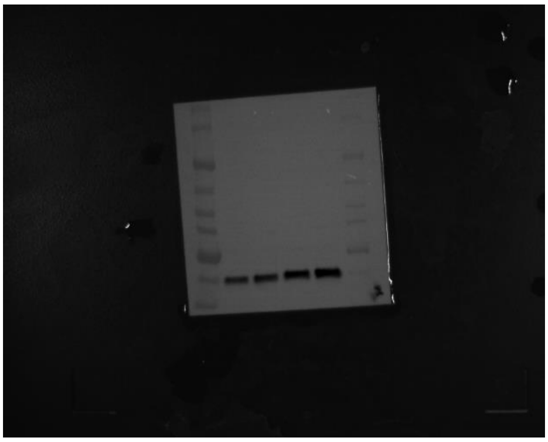

GAPDH

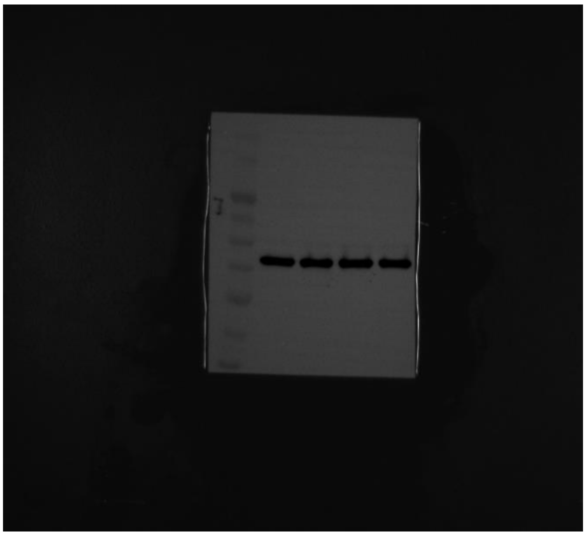

PAK1

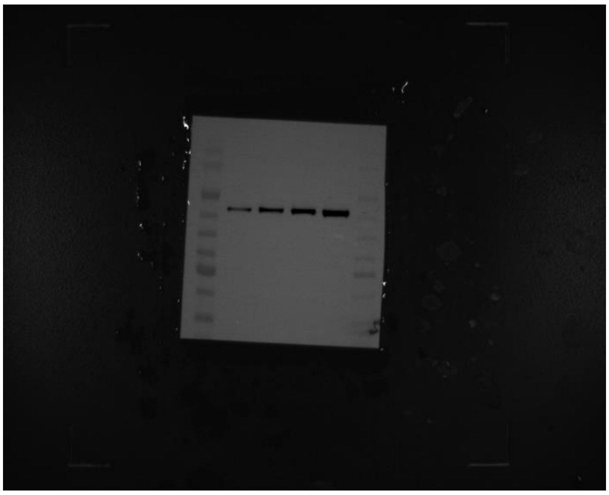

Paxillin

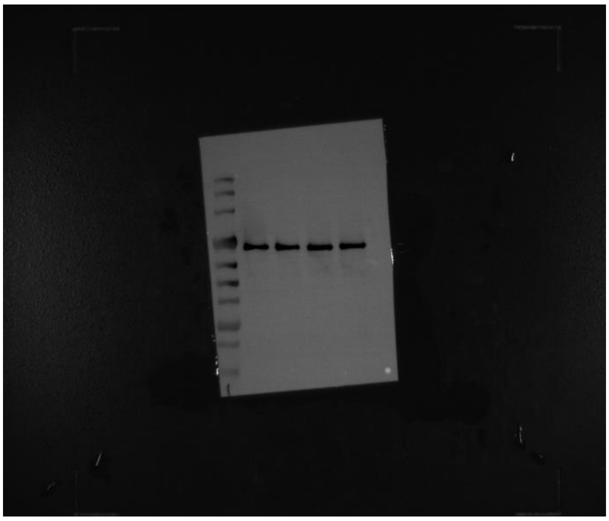

PY-31

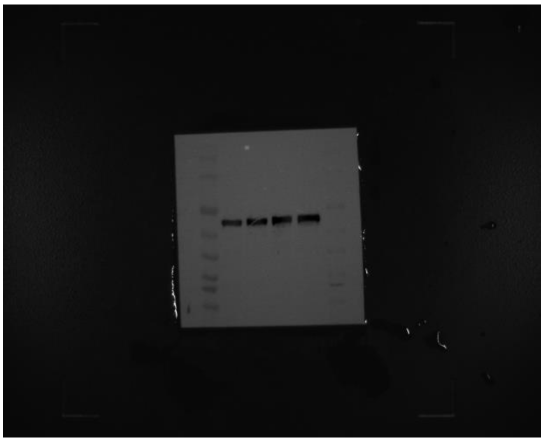

PY118

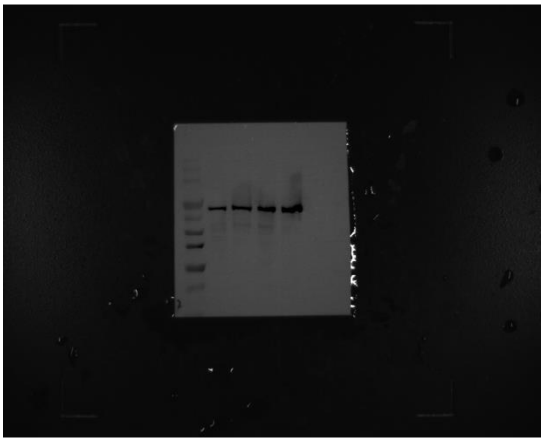

Figure 3-3B

CDC42

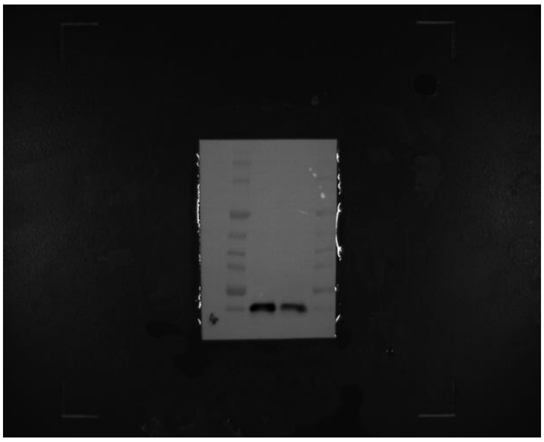

GAPDH

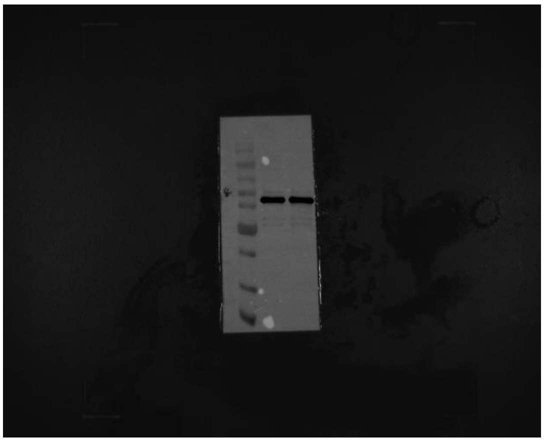

Figure 3-3E
eNOs

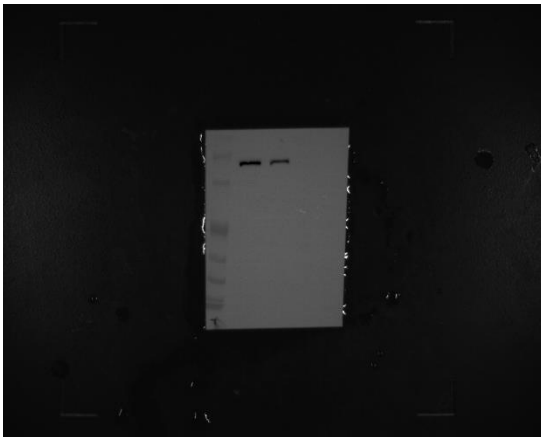

GAPDH

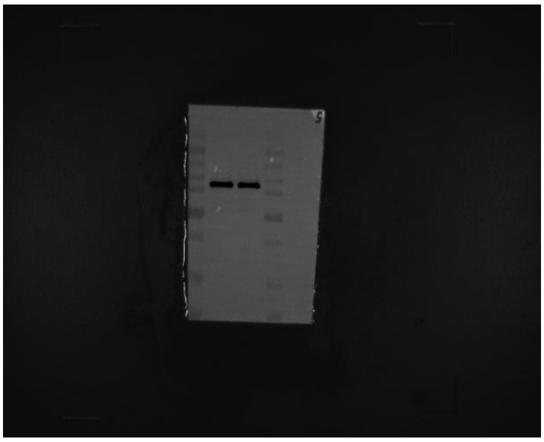

VE-cadhein

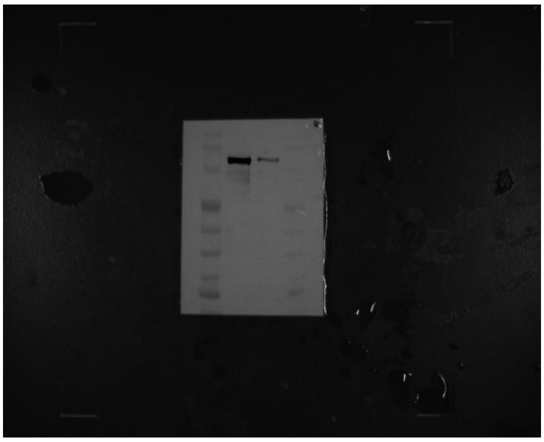

vWF

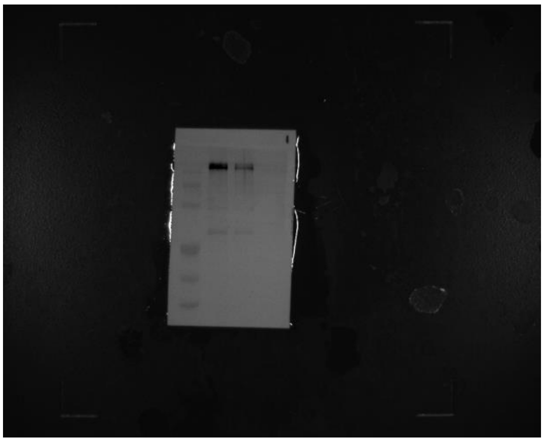

Figure 4-4A
CDC42

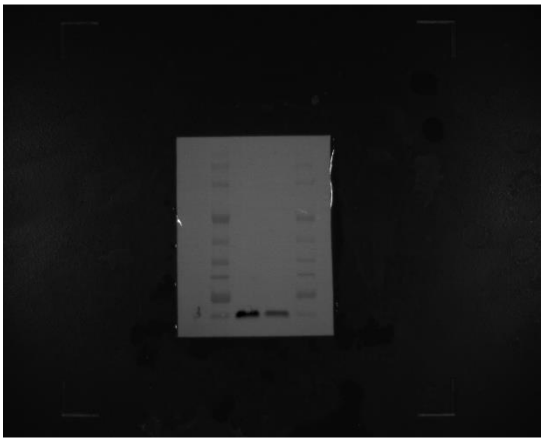

GAPDH

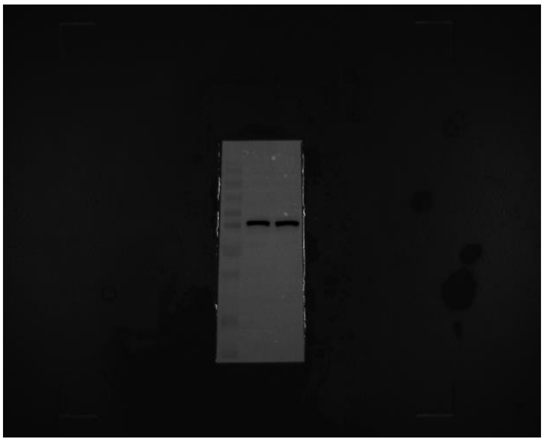

PAK1

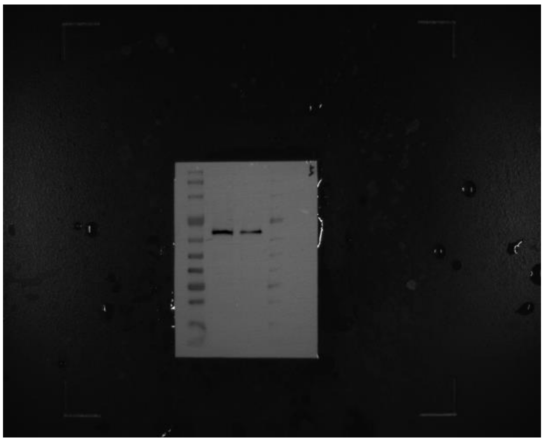

Paxillin

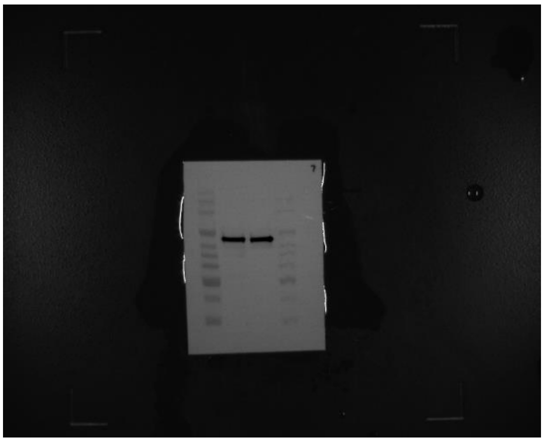

PY-31

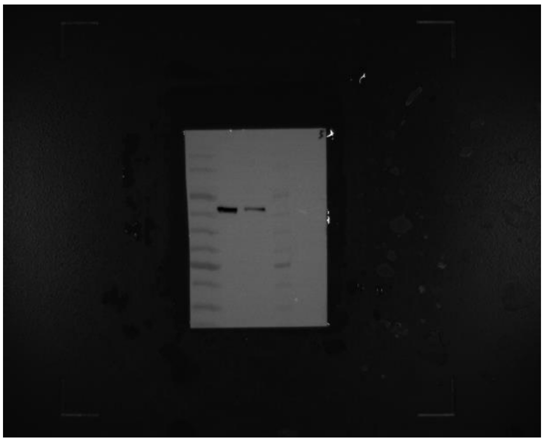

PY118

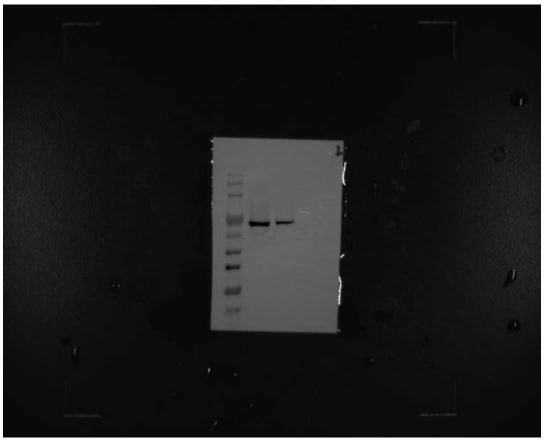

Figure 4-4B
eNOs

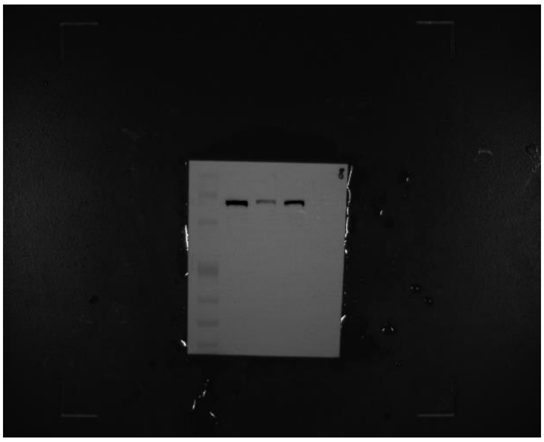

GAPDH

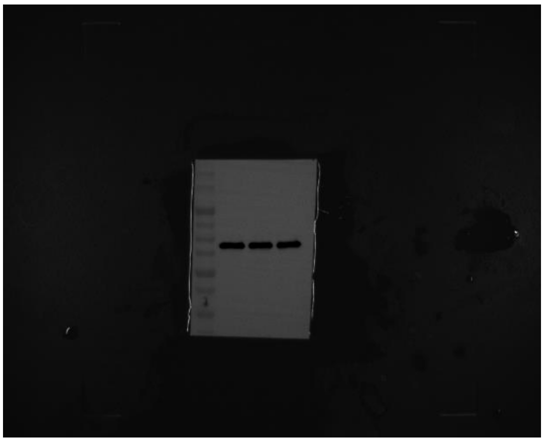

PAK1

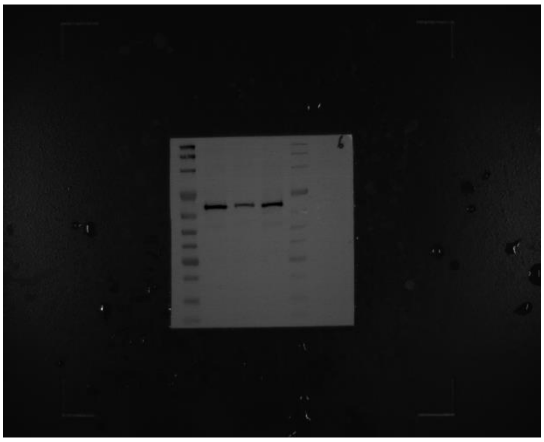

Paxillin

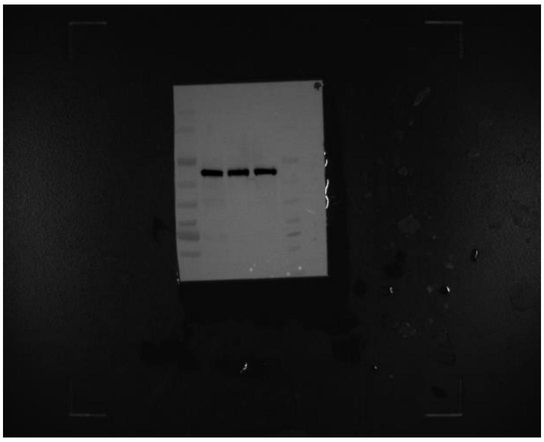

PY-31

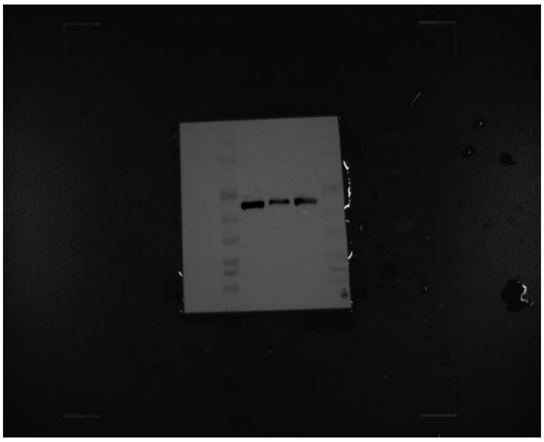

PY118

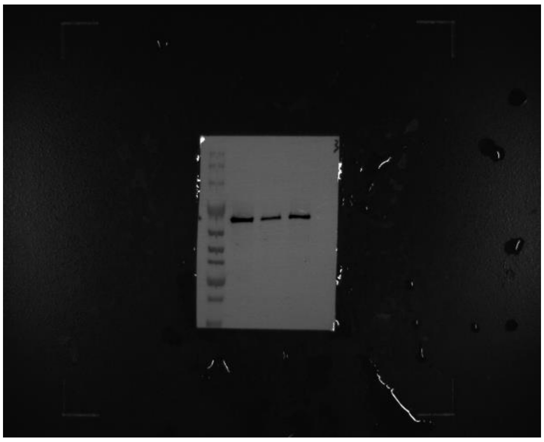

VE-cadhein

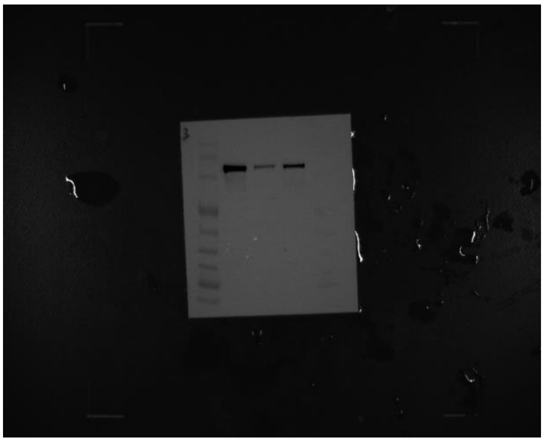

vWF

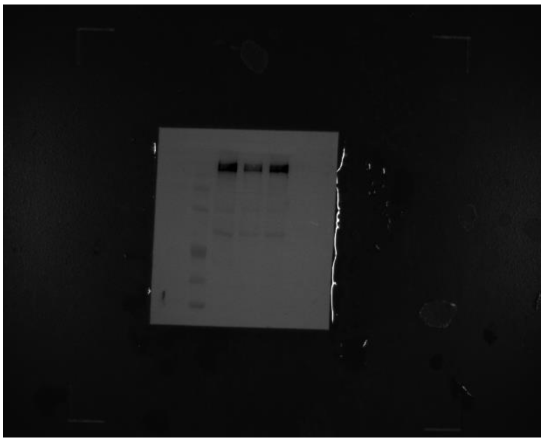

Figure 4-4D

eNOs

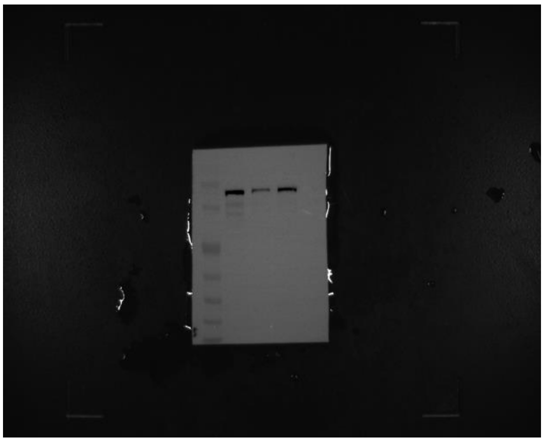

GAPDH

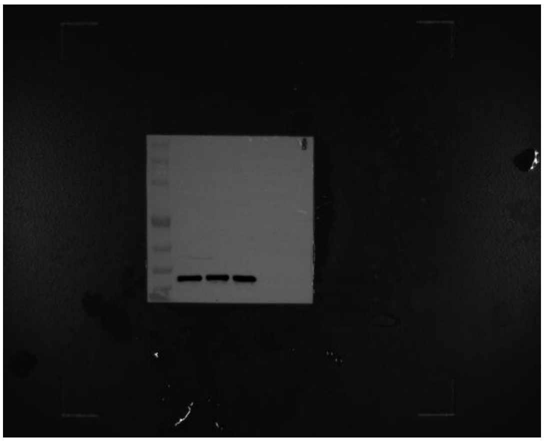

Paxillin

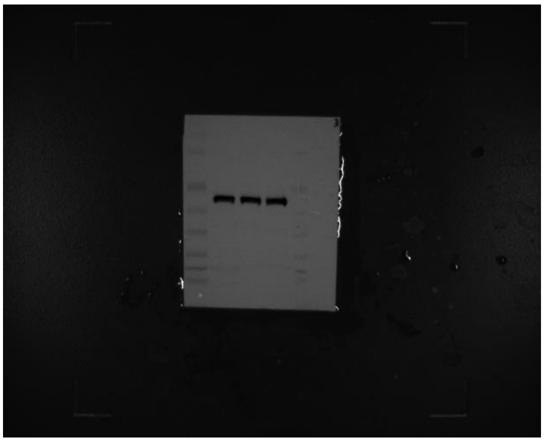

PY-31

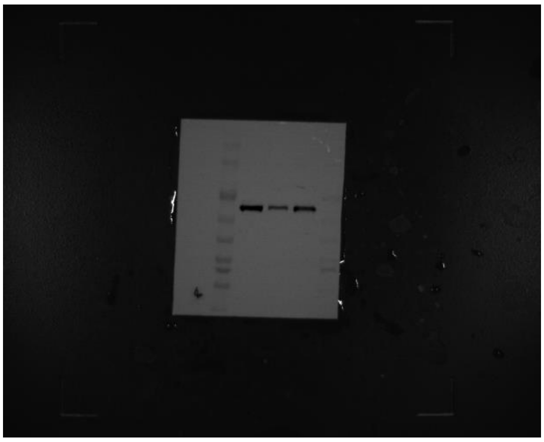

PY118

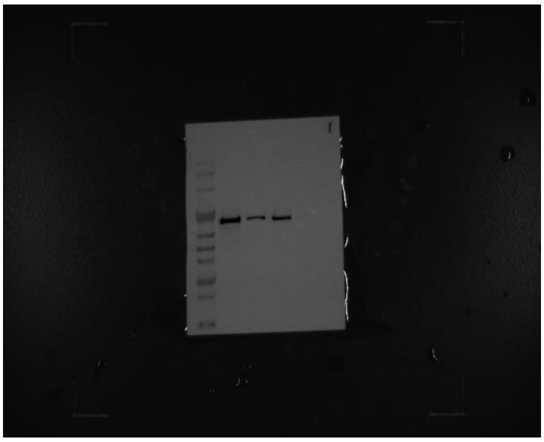

VE-cadhein

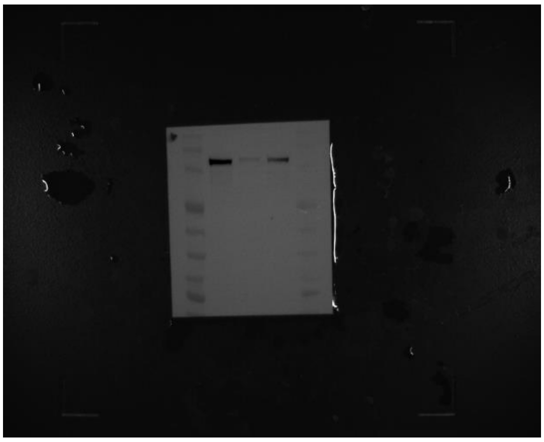

vWF

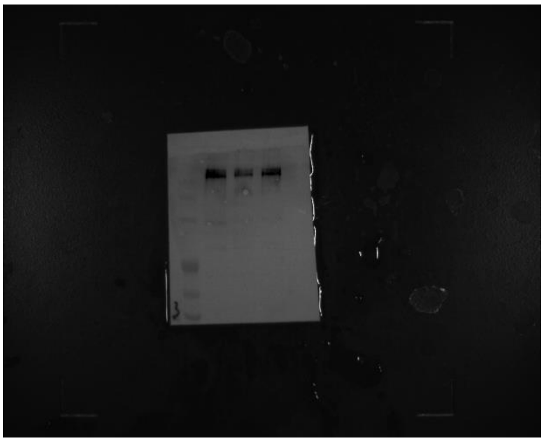

Figure 4-4E-1
CDC42 1-1

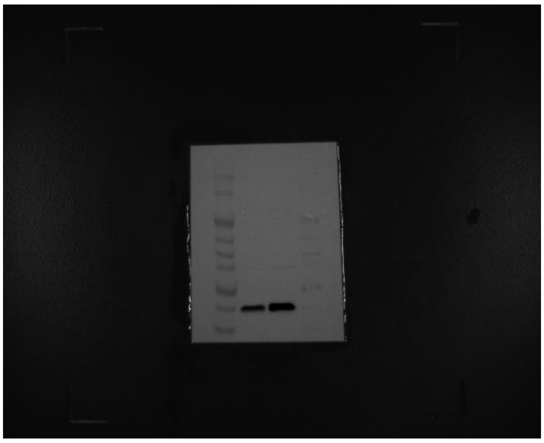

CDC42 1-2

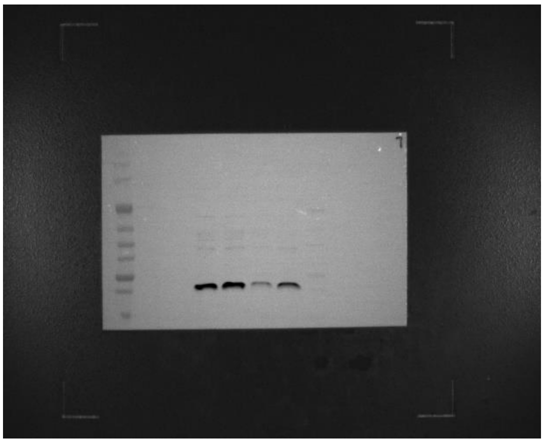

PAK1-1

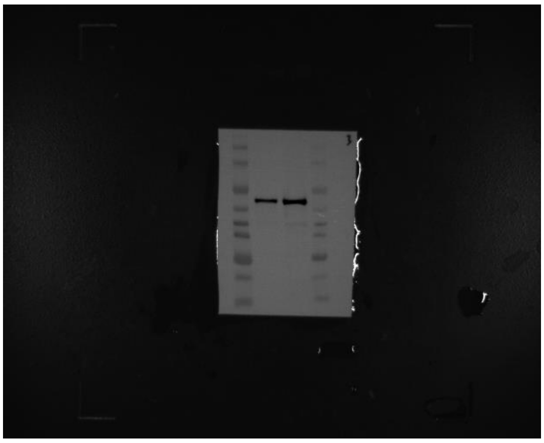

PAK1-2

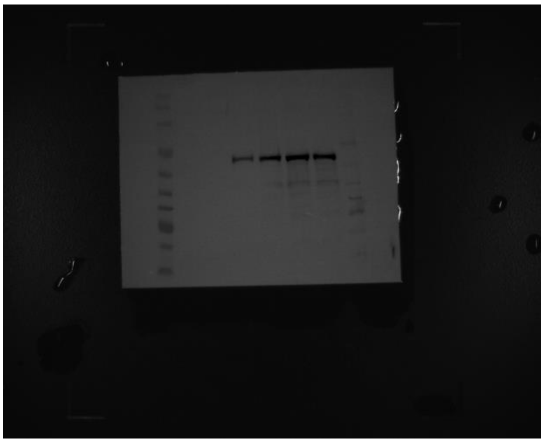

Figure 4-4E-2

CDC42 2-1

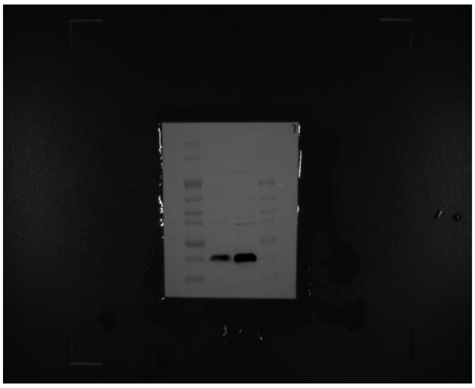

CDC42 2-2

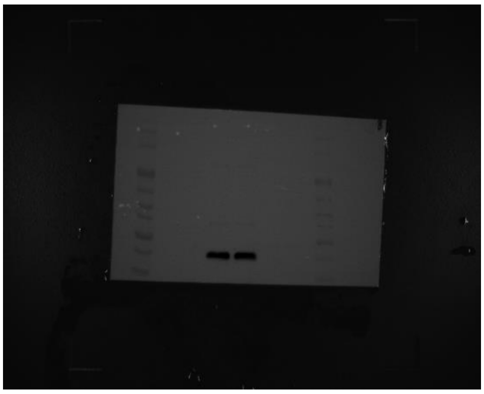

Paxillin 1-1

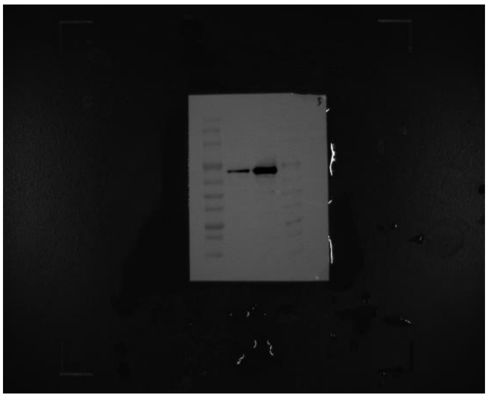

Paxillin 1-2

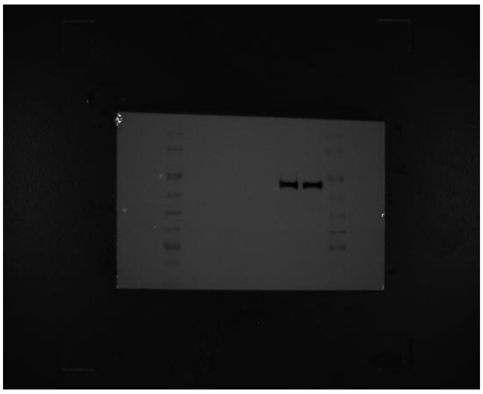

Figure 4-4E-3

PAK2-1

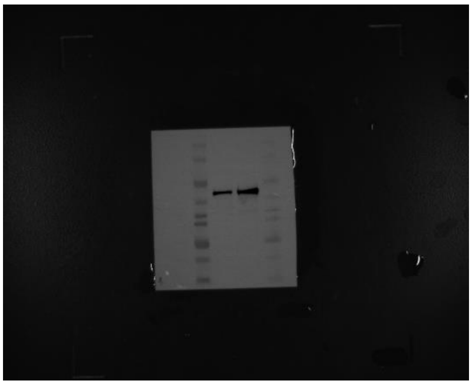

PAK2-2

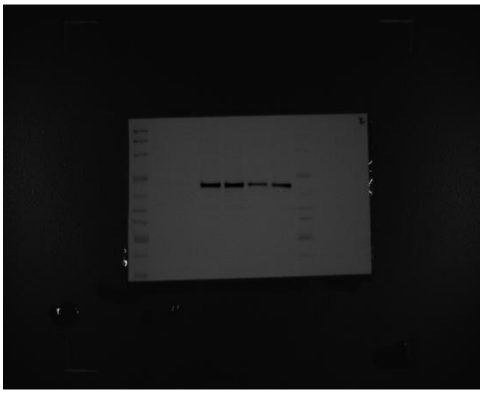

Paxillin 2-1

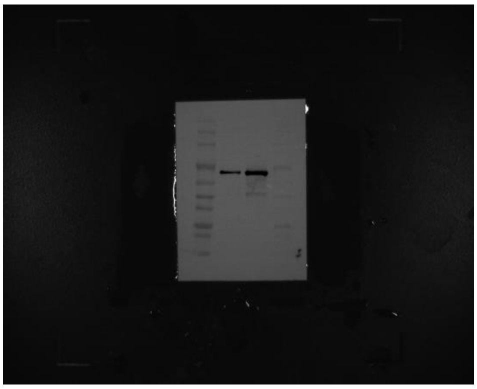

Paxillin 2-2

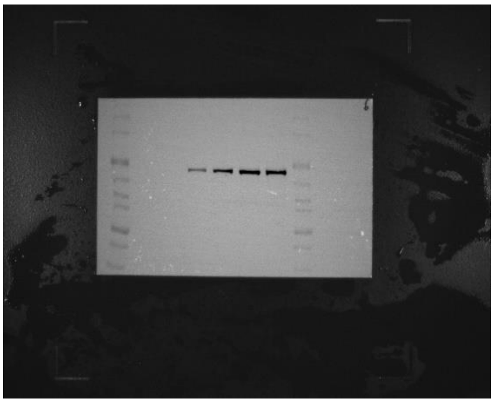

Figure 5-5C
CDC42

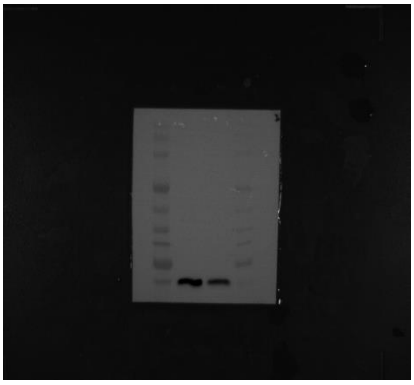

eNOs

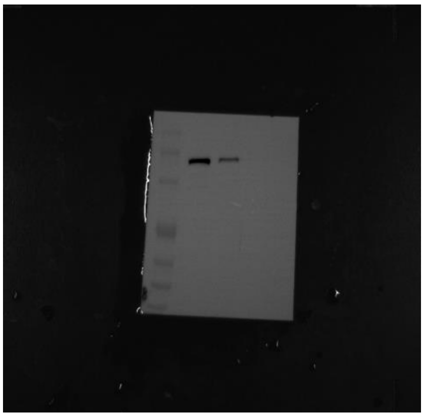

GAPDH

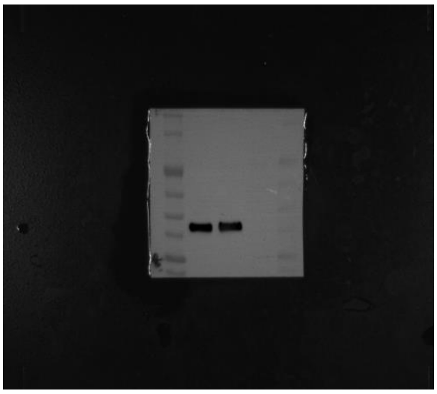

PAK1

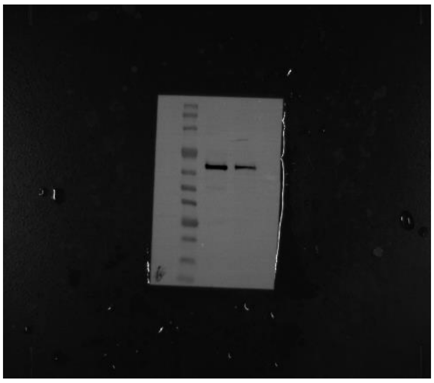

Paxillin

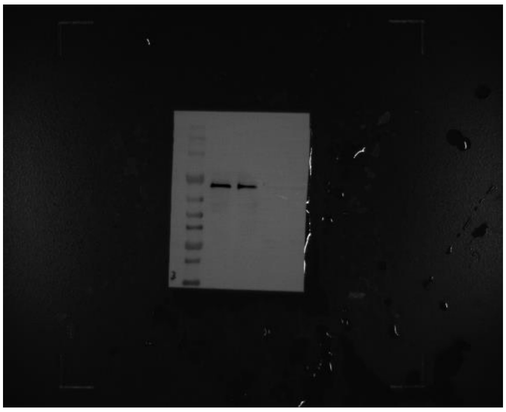

PY-31

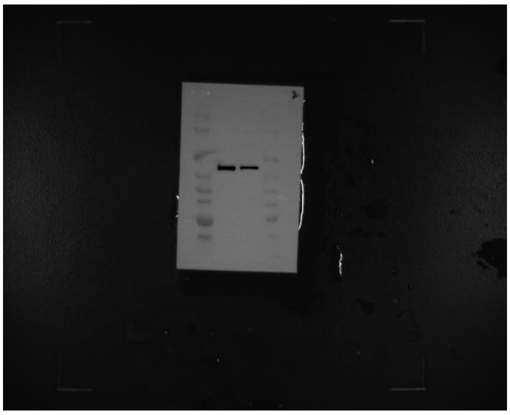

PY118

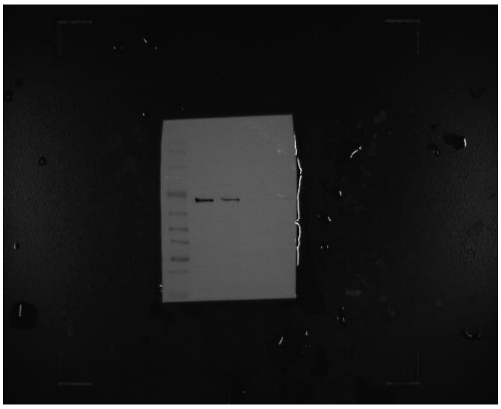

VE-cadhein

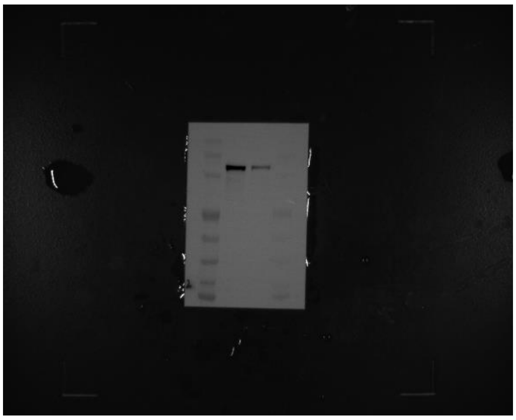

vWF

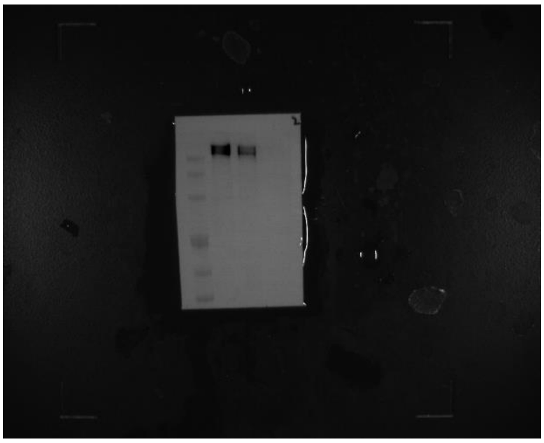

Figure 5-5H

CDC42

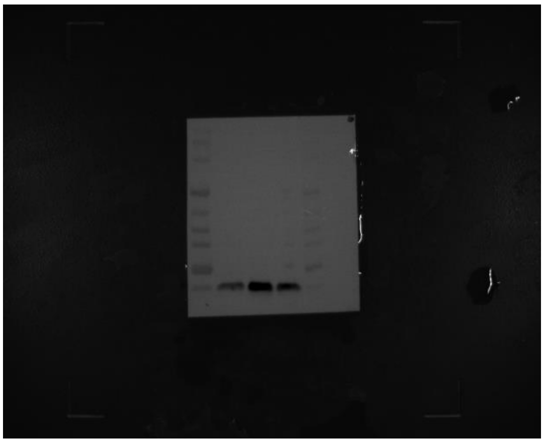

GAPDH

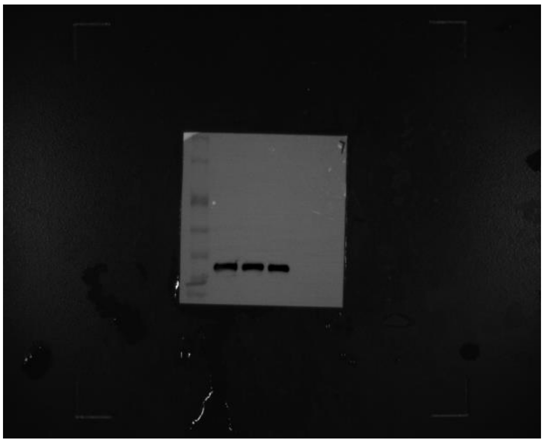

Figure 5-5H
CDC42

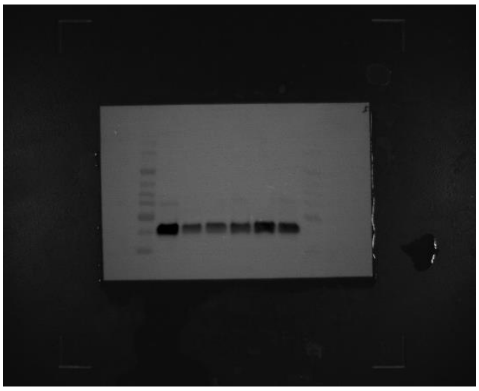

GAPDH

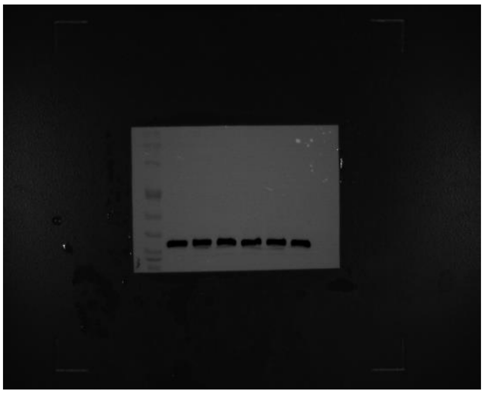

PAK1

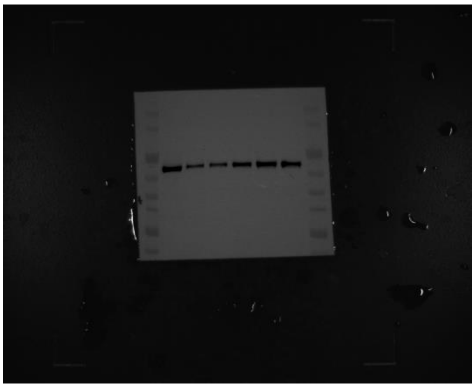

Paxillin

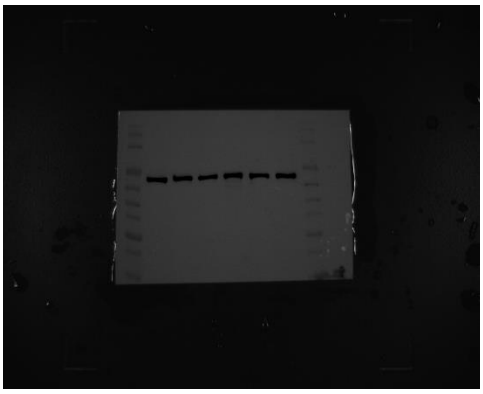

PY-31

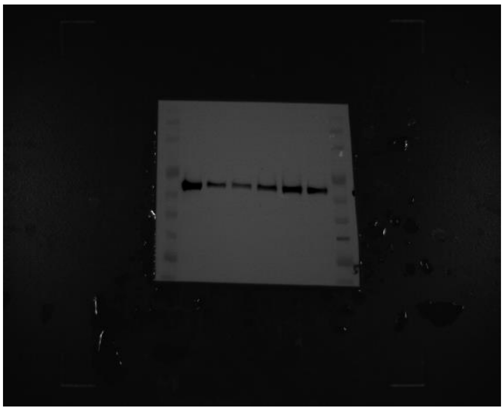

PY118

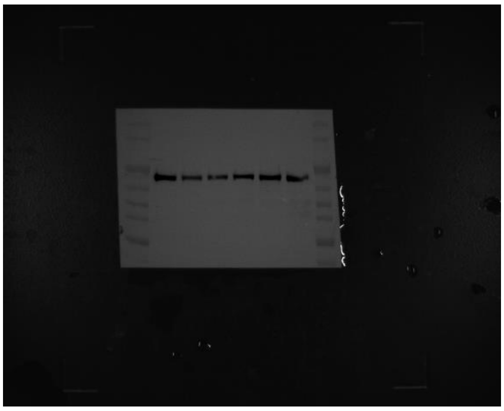

Supplement: Supplementary file 1 — Supplementary Material 1 [file 12958_2024_1240_MOESM1_ESM.pdf]
